# Avian Pathogenic *Escherichia coli*: Advances in Pathogenesis, Diagnosis, and Control

**DOI:** 10.3390/vetsci13010019

**Published:** 2025-12-24

**Authors:** Osama Kamal, Holger Kneuper, Tristan Cogan, Martin J Woodward

**Affiliations:** 1 Bristol Veterinary School, University of Bristol, Bristol BS40 5DU, UK; osama.kamal610@gmail.com (O.K.); tristan.cogan@bristol.ac.uk (T.C.); 2 Folium Science, Science Creates, Old Market, Bristol BS2 0JZ, UK; hk@foliumscience.com; 3 Department of Food Hygiene, Safety and Technology, Faculty of Veterinary Medicine, Zagazig University, Zagazig 44519, Egypt; 4 Department of Food and Nutritional Sciences, University of Reading, Whiteknights, Reading RG6 6AP, UK

**Keywords:** APEC, avian colibacillosis, CRISPR control, antimicrobial, endolysins

## Abstract

Avian colibacillosis is a significant disease in poultry caused by avian pathogenic *Escherichia coli* (APEC). APEC affects birds of all ages and leads to severe lesions in multiple organ systems, including the respiratory, digestive, and reproductive tracts. The disease imposes a substantial economic burden on poultry production due to increased mortality and decreased meat and egg production associated with the disease. Moreover, there is growing concern about the potential transmission of APEC to humans via contaminated poultry products, where it may cause extraintestinal infections. Given these implications for both animal welfare and public health, a thorough understanding of the pathogen is essential for developing effective control strategies. This review seeks to discuss the major facets of colibacillosis and outlines key considerations for advancing its diagnosis and effective management.

## Simple Summary

Avian pathogenic *Escherichia coli* (APEC) is the major etiological agent of avian colibacillosis, a condition that significantly compromises poultry health and productivity, adversely affects animal welfare, and leads to substantial economic losses worldwide. This review explores the organism’s characteristics, genetic diversity, pathogenesis, and virulence factors. Additionally, we highlight the limitations of conventional diagnostic and control approaches and discuss emerging advanced technologies that enhance diagnostic accuracy and offer promising new strategies for disease management.

## 1. Introduction

*Escherichia coli* is a Gram-negative bacterium that belongs to the Enterobacteriaceae family and known to be a normal resident of the intestinal tract of the vertebrate [1]. Although they usually present as a component of the normal intestinal biota, some strains have acquired the ability to cause disease and thus are classified as pathogenic [2]. Pathogenic *E. coli* generally differ from their non-pathogenic counterparts in possessing certain pathogenicity determinants or virulence factors that allow them to establish infection [3,4]. Based on their predilection for a site of infection, pathogenic *E. coli* can be broadly classified into two categories: those that act inside the intestinal tract, i.e., intestinal pathogenic *E. coli* (IPEC), or diarrheagenic *E. coli* (DEC) and others which extend beyond the intestinal barrier known as extraintestinal pathogenic *E. coli* (ExPEC) [5,6]. Deeper categorization is based on the virulence profile of the strain and the resulting clinical manifestation. For instance, enteroaggregative *E. coli* (EAEC), enterohaemorrhagic *E. coli* (EHEC), enteroinvasive *E. coli* (EIEC), enteropathogenic *E. coli* (EPEC), enterotoxigenic *E. coli* (ETEC) and diffusely adherent *E. coli* (DAEC) are all different pathotypes of the IPEC category [7,8].

Moreover, Clermont et al. clustered *E. coli* according to their genetic relatedness into seven phylogroups named A, B1, B2, C, D, E, and F [9,10], and, in a recent update, introduced a new intermediate phylogroup between the F and B2 phylogroups named the phylogroup G, expanding the total phylogroups to eight [11]. Virulent strains associated with extraintestinal infections usually belong to the phylogroups B2 and D [12,13]. On the other hand, the phylogroup A often contains the commensal *E. coli* [14]. APEC is a member of the extraintestinal category of pathogenic *E. coli* with potential to infect poultry of different ages and types, including broilers and layers, causing severe pathologies in different tissues collectively known as “avian colibacillosis”. The infection could be systemic resulting in airsacculitis, perihepatitis, pericarditis, peritonitis, and fatal septicemia [15]. Also, localized infections such as cellulitis and salpingitis could occur. The disease often results in high mortality and morbidity rates leading to severe economic losses. Furthermore, the zoonotic potential of APEC has been suggested, imposing a further burden on public health. This review focuses on different aspects of APEC including the pathogenesis of the disease, the involved virulence factors, and the zoonotic potential, and critically discusses the available control strategies.

## 2. APEC Pathogenesis and Pathological Picture

The widespread multiple lesions of avian colibacillosis affecting different organs indicate the systemic nature of the disease. The chicken gut constitutes a complex set of niches for a variety of bacterial species including *E. coli* [16,17,18]. The *E. coli* in the intestine of healthy chicken could have multiple virulence factors, rendering them as potential APEC able to initiate infection [19,20]. Being residents of the intestinal tract allows potential APEC to spread via chicken debris into the surrounding environment [21]. This highlights the role of free-living birds in the transmission of the disease. However, for intensive farming, bad housing conditions exacerbate the situation by increasing the *E. coli* counts in the surrounding environment with a bacterial load in the poultry house dust up to 106 CFU of *E. coli* per gram [15,22]. In turn, this constitutes a potential source of infection to chicken through contaminated feed, water, and equipment, as well as aerosol and dust [23].

**Figure 1:**
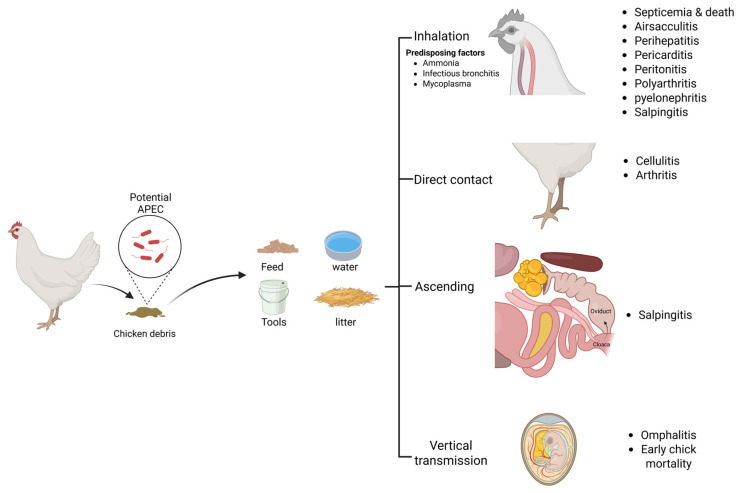
Pathogenesis of APEC: transmission routes and lesions.

APEC can initiate either primary or secondary infection [24]. The pathogen can transmit to healthy birds either horizontally or vertically (Figure 1). The horizontal transmission is mainly through the respiratory route and usually results in the generalized form of the disease [25,26,27]. However, other routes of transmission that result in localized forms of the disease also occur, such as the ascending route from the cloaca to the oviduct leading to salpingitis [28,29], and the direct contact through skin leading to cellulitis [30]. APEC are also able to transmit vertically from diseased breeders to the yolk sac [29,31]. Therefore, the outcome of infection depends mainly on the route of transmission and the immune status of the bird. Several factors were found to predispose for APEC infection, such as elevated ammonia levels in the poultry house and the infection by other respiratory pathogens such as Mycoplasma and infectious bronchitis (IB) [32]. Ammonia is toxic and is known to damage epithelial cells, enabling APEC infection via the air sac and lung [32,33].

As mentioned above, the generalized (systemic) form of colibacillosis usually occurs through the respiratory route. Once the bacterium is inhaled, it establishes itself in the respiratory tract via adhesion to the lining epithelium [34,35]. This requires the bacteria to overcome the innate defensive barriers such as the lining mucous and the existing antimicrobial peptides [36]. The contact between APEC and the epithelial cells of the respiratory tract stimulates regulatory changes of the cells such as the upregulation of the toll-like receptors in lung and tracheal cells [33,37]. The toll-like receptors mediate pathogen recognition and pro-inflammatory cytokines production such as IL-1B, IL6, and IL8 [38,39]. The cytokines stimulate the chemotaxis of innate immune cells such as macrophages and heterophils to the site of infection [40]. A definitive characteristic of the avian lung-associated immunity is the scarcity of the resident immune cells such as heterophils and macrophages on the lining epithelium [41,42]. This feature, besides the large surface area and the very thin blood–gas barrier of the bird’s lower respiratory tract, may increase the susceptibility to infection by respiratory pathogens [43]. Innate immune cells such as heterophils and macrophages were found to increase in the lungs and spleen after experimental infection of chickens with APEC [44,45,46]. This indicates that the defense against bacterial infection in chicken lungs is mainly dependent on the recruitment of blood immune cells to the site of infection [33]. Alber et al. reported a higher heterophil than macrophage population in the lung six hours post inoculation with two different APEC serotypes, which suggests heterophils as the main early responder at the site of infection [46]. Both macrophages and heterophils are phagocytic cells that engulf the pathogen to contain the infection with additional antigen-presenting features for macrophages [47]. Upon activation of the antigen-presenting cells, the chemotaxis of more immune cells to the site of infection takes place mediated by cytokines [48,49,50]. Simultaneously with the activation of macrophages, other adaptive immune cells are activated, such as CD4+ T cells that recognize the presented antigen which indicates the synergy between the innate and the adaptive immune responses in the fight against APEC [50,51].

The immune response in the lungs of infected bird results in inflammation of the respiratory tissues such as air sacs and bronchi (i.e., airsacculitis and bronchitis, respectively), with congestion of blood capillaries, oedema, fibrinous deposits, and inflammatory cell infiltrations in the air sacs [27]. Loss of air sac transparency with yellowish white exudate could be seen. Despite the inflammatory response, APEC have multiple virulence factors that help the bacterium to overcome immunity and evade phagocytosis such as the capsule, fimbriae, and the surface LPS [22,52]. Furthermore, APEC were reported to invade the cells of the respiratory epithelium and the adjacent fibroblasts [35,53]. They are also able to actively invade, survive, and proliferate inside macrophages [46,52,54,55], or even induce apoptosis of the macrophages [38,56]. The invasive nature of APEC into the epithelia and phagocytes might explain the detection of the pathogen in the blood stream of the bird a few hours post inoculation through the trachea or air sac and accounts for the generalized pathological picture that follows the localized lesions in the respiratory tract [26,46,56]. However, the precise molecular mechanism of invasion by APEC still needs further studies. Once the bacterium reaches the circulatory system, the systemic form of infection is established. Systemically infected birds show lethargy and a decrease in water and feed intake, followed by sudden death due to septicemia [15]. The postmortem picture of the dead birds usually includes airsacculitis and inflammation of serous membranes, i.e., perihepatitis, pericarditis, and peritonitis that manifest as congested blood capillaries and fibrinous layers deposited on the liver and heart [57,58,59]. Moreover, other gross lesions are frequently encountered with the systemic form of colibacillosis, such as an enlarged and congested spleen, pyelonephritis, polyarthritis, and salpingitis [15,27,60].

Alternatively, salpingitis and cellulitis are two frequently encountered localized forms of colibacillosis. Salpingitis could occur as a consequence of the systemic course of the infection or as an ascending infection from the cloaca to the oviduct, leading to contaminating the egg yolk, which could be exacerbated into egg peritonitis if the yolk is released into the coelomic cavity, resulting in inflammation of the peritoneum [60,61]. Salpingitis has a drastic effect on egg productivity in layers, and also can result in mortalities of early-life chicks [15,60,61]. On the other hand, cellulitis usually results from infection through injured skin, severely impacting carcass quality by subcutaneous lesions and leading to a high rate of carcass disposal at abattoirs [15,60,62]. It is worth noting that the newly hatched chicks could be infected via vertical transmission from diseased breeders or through contaminated eggshell, leading to omphalitis and high mortality rates during the first week after hatching [29,63,64,65]. The overall outcomes of avian colibacillosis, including the high mortality rates of chicks, the reduced performance and meat production of broilers, the reduced egg production of layers, and the increased rate of carcass condemnation at abattoirs, account for the significant economic loss of the poultry industry associated with the disease.

## 3. Virulence Factors

The virulence factors described in this section are those for which strong evidence for their role during APEC pathogenesis has been gained.

### 3.1. Adherence

The first step of the pathogenesis of an infection is the attachment of the pathogen to the epithelial cells at the specific site of infection, typically mediated by adhesins. These are membrane proteins located on the bacterial surface that recognize and bind to certain receptors on the host cells [66]. Through this adhesin–receptor interaction, bacteria can colonize different host tissues either to initiate an infection or persist as a part of the commensal microbiota [67]. APEC express diverse adhesins that support their infection process [68]. Among these, type 1 fimbriae are the most commonly expressed and play a key role in colonizing the chicken respiratory tract. This fimbrial structure is produced by the fim gene cluster, which encodes the fimA, fimC, fimD, fimF, fimG, and fimH subunits. These subunits assemble into a proteinaceous appendage on the outer membrane of the bacterial cell that facilitates bacterial attachment to specific host cell receptors [69].

Type 1 fimbriae are mannose-sensitive, requiring mannose-containing receptors for effective binding [68]. APEC strains lacking type 1 fimbria exhibit reduced adherence abilities [70]. These fimbriae enable APEC to attach to tracheal epithelial cells during the early stages of infection [68,71]. In addition to mediating adherence, type 1 fimbriae aid immune evasion. Specifically, the FimH adhesin at the fimbrial tip binds to CD48 receptors on phagocytes, promoting internalization into macrophages. Within macrophages, vesicles containing FimH-positive bacteria supress the release of free radicals preventing vesicle acidification and helping the bacteria to survive inside the phagocyte [72]. Furthermore, these fimbriae may contribute to the antimicrobial resistance of APEC by enhancing bacterial internalisation and persistence within macrophages [73].

APEC also express another fimbrial adhesin: the P fimbriae [74]. Encoded by the pap operon—a cluster of eleven genes located on a pathogenicity island on the bacterial chromosome—these structures feature the PapG adhesin, which binds to glycolipid receptors containing Galα(1–4)Gal residues [75]. While being commonly distributed in *E. coli* strains associated with urinary tract infection (UTI) in humans [76,77], pap operon genes have also been frequently identified in the pathogenic *E. coli* from poultry [78,79,80]. Deletion of these genes significantly attenuates APEC virulence compared to wildtype strains [81]. P fimbriae are not typically expressed by APEC in the upper respiratory tract compared to air sacs and lungs, which suggests the role of such fimbriae during the advanced stages of APEC infection [68].

### 3.2. Iron Acquisition Systems

Iron is an indispensable element for all living organisms, including bacteria, essential for cell composition, growth, multiplication, and metabolism [82,83]. Bound iron is usually present in host’s biological fluids either included in hemoglobin or bound to certain carriers such as transferrin [83,84]. In addition, in aerobic environments, iron is typically found in its oxidized ferric form, which has low solubility and therefore limits its availability [84]. Under the pressure imposed by the iron-restricted environment of the animal body, bacteria face the challenge of obtaining iron required for their survival. To overcome iron limitation, bacteria secrete small molecules called siderophores that chelate and solubilise iron to be available for bacterial use [85,86]. Siderophores are important for bacterial pathogenesis. Reduced virulence was reported with strains that had a defective siderophore production [87]. *E. coli* can secrete a variety of siderophores, including aerobactin, salmochelin, and yersiniabactin [86,88]. An operon of five genes (*iucD*, *B*, *A*, *C*, and *iutA*) typically harboured on the colV plasmid is responsible for aerobactin synthesis in *E. coli* [89,90,91].

An APEC strain individually knocked out of the aerobactin genes showed a reduction in pathogenicity evidenced by reduced colonization and competitive growth with the wildtype strain, which indicates the importance of the aerobactin siderophore for APEC virulence [92,93]. Yersiniabactin, another key siderophore, was initially identified in *Yersinia* spp. It is encoded on a high pathogenicity island (HPI) by seven genes, all required for the synthesis of the siderophore [94,95,96]. The most notable of these genes are the *irp1* and *irp2* genes [97,98]. Generally, the *Yersinia* HPI is associated with increased virulence in ExPEC [99,100]. Blocking the uptake of the yersiniabactin by deleting the gene responsible for its receptor from an ExPEC strain resulted in virulence attenuation [101]. Furthermore, deleting the core HPI gene (*irp2*) or the yersiniabactin receptor gene (*fuyA*) reduced the virulence of the APEC knockout strains evidenced by attenuated adherence compared to the wildtype [102]. Salmochelin is another siderophore secreted by *E. coli*, encoded by the *iroBCDEN* gene cluster and found on virulence plasmids of the ExPEC [103,104]. The *iroN* gene encodes the outer membrane receptor for the salmochelin siderophore [105]. This gene, along with other iron receptor genes, is highly prevalent in APEC [106,107]. The absence of *iroC*, *iroD*, or *iroN* genes impaired the virulence of an APEC O78 strain [108,109]. The iroN receptor protein on its own was found to enhance biofilm formation in ExPEC [110]. Comparative studies show that iron acquisition systems are more frequently detected in APEC strains than in commensal *E. coli* [111]. These systems are important for pathogenic *E. coli* to establish infection by providing a competitive advantage in iron-restricted environments [109]. The possession of more than one iron-acquisition system by APEC appears to potentiate the bacterial virulence and survival [79].

### 3.3. Increased Serum Survival

Due to the invasive nature of APEC, it is necessary for the bacteria to survive in chicken serum to be able to establish a generalized infection. One important factor reported to significantly increase APEC resistance to chicken serum is the increased serum survival protein, Iss [112,113,114]. Iss is an outer membrane protein encoded by the *iss* gene, typically located on the ColV plasmid [114,115,116]. Previous studies have reported the prevalence of the *iss* gene in *E. coli* strains isolated from septicaemic birds [117,118,119]. The gene has also been identified in other ExPEC strains including the uropathogenic *E. coli* (UPEC) and newborn meningitis-causing *E. coli* (NMEC) [120,121]. In one-day-old chicks, the presence of the iss gene resulted in a 20-times increase in serum resistance compared with other isogenic strains lacking this gene [115,122]. Although the mechanism of action of the Iss protein in protecting APEC in serum is not fully understood, it has been reported that the Iss protein is required for the synthesis of the group 4 capsule that envelops the bacterial cell and protects its membrane from the action of the membrane attack complex (MAC), helping the bacteria to survive in the serum [114,123]. Given its role in virulence and its strong association with ExPEC, and particularly with APEC, the presence of the *iss* gene represents a reliable marker for APEC identification [124].

Other than the virulence factors already described in this section, several additional putative virulence genes have been identified, for which confirmed roles in pathogenesis are less well defined. However, these genes have proven valuable in diagnostic screening and in the differentiation of APEC isolates, as their prevalence suggests an association with pathogenesis rather than a confirmed causal role (see the Diagnosis Section 5 below). Further information on other virulence-associated genes can be found in ref. [31].

## 4. APEC Zoonosis

In the context of the One Health concept, understanding the dynamics of interspecies transmission of pathogens is crucial. Animals constitute a source of a wide range of human pathogens, including *E. coli*. For instance, it is well established that cattle are the reservoir of the enterohaemorrhagic *E. coli* (EHEC) O157, a strain that causes severe illness in humans while remaining harmless to the bovine host [125]. Moreover, poultry is also suggested as a probable source of ExPEC infection in humans, either via direct contact with birds or consumption of contaminated chicken products [126]. This association is supported by cumulative evidence of similarity between APEC and human UPEC and NMEC strains. Early studies highlighted the overlap in serotypes and virulence genotypes among *E. coli* strains isolated from diseased birds and human clinical cases [127,128]. Moreover, a number of fecal strains from healthy chicken and their environment exhibited virulence profiles resembling both outbreak-associated APEC and human ExPEC strains, suggesting the poultry intestine and their environment as potential sources of APEC and human ExPEC infections [21].

Other studies touched on more discriminatory approaches such as phylogenetic analysis of APEC and human ExPEC based on MLST and PCR-based phylogeny. Such studies showed host independence of clonal clustering of the strains with phylogroups composed of poultry and human isolates sharing the same traits [129,130]. In addition, findings from phylogenetic studies directed the attention to certain sequence types or clonal complexes as of a significant zoonotic potential such as clonal complexes ST131, ST95, ST23, ST117, and ST45 [131,132,133,134]. Most of these prominent ExPEC sequence types have been detected in poultry meat, suggesting a possible route of transmission to humans through the food chain [135,136]. Notably, some human ExPEC strains sharing the same phylogenetic subclusters and nearly the same virulence factors as poultry strains showed lethality to one-day-old chicks [137,138]. Conversely, strains of poultry origin were pathogenic to mouse models and infective to human tissue culture, which supports their potential pathogenicity to humans [19,139,140,141,142,143].

Advanced genome sequencing technologies with extensive in silico analysis have enabled deeper investigations into the genetic relatedness of bacterial isolates, uncovering relationships that might be overlooked using the classical phylogenetic methods such as ordinary MLST. The first genome sequence of an APEC strain (O1:K1:H7) was completed by Johnson et al. and revealed a significant similarity to ExPEC genomes [144]. A core genome MLST (cgMLST) study analyzing 2547 alleles that constitute the core genome of *E. coli* strains under study instead of the usual seven housekeeping genes revealed the relatedness of the ST131 strains collected from different sources, including humans, chicken, and chicken products [145]. Even higher resolution is achieved through single nucleotide polymorphism (SNP) analysis of whole-genome sequencing (WGS) data. For instance, Pietsch et al. identified significant genomic similarity among three *E. coli* isolates—one from poultry and two from human patients—based on SNP comparisons [145]. Additionally, WGS has reinforced the zoonotic relevance of specific APEC sequence types such as ST131 [146,147], ST95 [148], and ST117 [149], which are increasingly associated with extraintestinal infections in humans. Although direct evidence linking APEC to human infection outbreaks remains unavailable, the growing body of research supports their zoonotic potential, resting on three main pillars: (a) shared characteristics between human and poultry isolates—including serotypes, virulence factors, and resistance genes; (b) demonstrated genetic relatedness through high-resolution genomic tools; and (c) cross-species infectivity shown in animal model experiments.

## 5. Diagnosis and Identification Scheme

Despite the well-characterized picture of the disease, identification of the APEC pathotype is always a complicated process due to the genetic diversity of the pathogen [150]. Traditionally, serotyping and virulence genotyping were used to identify APEC isolates [30,151]. Serotyping is based on identification of the somatic O-antigen either via PCR or specific antibodies. The use of serotyping for APEC identification stemmed from the observation that certain *E. coli* serotypes such as O78, O1, and O2 were the most prevalent among *E. coli* isolated obtained from diseased birds [112,152,153]. Besides serotyping, several studies aimed at linking APEC to certain virulence genotypes. Several virulence determinants are required for APEC to establish infection and cause pathology in the avian host [154]. Some of these factors are important for epithelial adhesion and others are related to iron acquisition and serum resistance [117,154,155].

However, the same pattern of virulence factors is not always present in all pathogenic strains associated with any one clinical manifestation [156,157]. Usually, variable combinations of genes are found, which complicates the consideration of a certain virulence gene or gene set as the definitive pathogenicity determinant [158,159,160,161,162]. Previous studies suggested combinations of 4 to 10 genes as indicative of the APEC genotype. Combinations of four genes (*iss*, *tsh*, *iucC*, and *cvi*) [163], five genes (*hlyF*, *iss*, *iutA*, *ompT* and *iroN*) [124], six genes (*iss*, *iucD*, *hylF*, *ompT*, *iroN* and *iutA*) [161], eight genes (*astA*, *vat*, *iss*, *irp2*, *iucD*, *papC*, *tsh*, and *cva/cvi*) [164], and ten genes (*iss*, *iutA*, *cvaC*, *iroN*, *fyuA*, *irp2*, *sitA*, *tsh*, *ompT* and *hlyF*) [165] were all suggested for the identification of APEC. Furthermore, a recent comprehensive review suggested a compilation of ten virulence determinants, e.g., *iss*, *tsh*, *iroN*, *ompT*, *iutA*, *cvaC*, *hlyF*, *iucD*, *papG*, and *papC*, as an identifier of the potential APEC pathotype [166]. It is worth noting that using any of the previous combinations as the exclusive indicator of the APEC genotype is inadequate. Many APEC isolates recovered from diseased birds were positive for none of the aforementioned genes [16]. Equally, many of those virulence genes were found in commensal isolates from healthy birds [156,162,167]. Those could be referred to as potentially virulent or opportunistic, which may cause an infection upon immune suppression by environmental stressors or any other factors [19,156]. This dilemma was discussed in a recent study conducted by Kazimierczak et al., who combined in silico virulence genotyping with an in ovo embryonic lethality test to verify the pathogenicity of the strain collection under the study [168]. Commensal strains that were categorized as non-pathogenic depending on the source and the original Clermont classification were found pathogenic after the in ovo embryonic lethality test was included. The study suggested three genes, i.e., *iroC* and *hlyF* and the *wzx* gene encoding the O antigen flippase of the O78 serotype, as predictors of the APEC pathotype. However, the test had low specificity (64.71%) [168].

Although PCR-based scanning of virulence genes is an easy and useful method for identifying APEC, most of these genes are plasmid-borne [169,170], which are mobilizable between bacteria, leading to fluctuating prevalence of these genes across bacterial populations, making it difficult to rely solely on them for accurate APEC identification. Also, it was reported that the pathogenicity of *E. coli* in chicken is irrespective of plasmid carriage and more related to clonal relatedness with certain lineages such as ST23, ST131, and ST117 are dominating the pathotype [171]. This, in turn, reflects the necessity of a deeper investigation of the genetic features associated with APEC based on more stable and conserved chromosomal markers. Whole-genome sequencing (WGS) technologies offer a high-throughput approach for analyzing large numbers of APEC isolates, providing comprehensive data on serotypes, sequence types, antimicrobial resistance genes, virulence factors, and phylogenetic relationships [172]. This approach has greatly enhanced our understanding of the genetic diversity among APEC serotypes and sequence types commonly associated with clinical disease [173,174,175].

Analysis of WGS data derived from APEC O78 isolates allowed the separation of this serotype into two phylogenetically distinct lineages, i.e., ST23 clonal complex and ST117, that belong to two different phylogroups, i.e., C and G respectively [30]. This underscores the high resolution of WGS technologies, enabling deeper identification of APEC beyond traditional serotyping [30]. Moreover, genomic data allowed for the clustering of APEC based on single nucleotide differences—i.e., single nucleotide polymorphisms (SNPs)—which further subdivided phylogenetic clusters into subclusters [176]. Despite the deeper clustering of APEC provided via WGS, the association of a certain virulence profile with a specific phylogroup or sequence type or even with a certain lesion—such as acute or chronic salpingitis—remains, as yet, still unobtainable [30,170,177]. For instance, a study that linked virulence factors to certain phylogroups such as B1 and A [178] was contradicted by another study conducted by Chen et al. that associated virulence genes with different phylogroups such as B2, D, and F [175]. Therefore, a more precise comparison of *E. coli* WGS data from chickens—beyond standard phylogenetic analysis and presence/absence screening of virulence determinants—could aid in identifying characteristic pathogenic traits. An extensive genome-wide association study (GWAS) utilized the pangenome of *E. coli* isolated from healthy and symptomatic birds to investigate allelic variation in core and accessory genes, independent of their clonal relatedness [179]. Interestingly, the study reported the shortage of phylogeny and plasmid-associated gene screening for pathotype identification and suggested, via the GWAS approach, a panel of 143 genes involved in different functions in *E. coli*, such as heat shock response, lipopolysaccharide synthesis, metabolism, antimicrobial resistance, and toxicity, as pathogenicity-associated genes [179]. This highlights the substantial contribution of WGS studies to understand APEC and expands our vision about the definitive characteristics of the pathogen beyond its virulome.

## 6. Control Strategies of APEC

Due to the significant health and economic impact of APEC, various control strategies have been deployed to control the pathogen and limit the burdens of the disease. Managemental measures, vaccination, antibiotics, and antimicrobial alternatives such as probiotics and bacteriophages are used to control, with varying degrees of success, the disease on poultry farms.

### 6.1. Management and Biosecurity Measures

As a first step in reducing the incidence of colibacillosis on poultry farms, addressing the predisposing factors of the disease is essential. These factors may include biological causes, such as primary viral infections like infectious bronchitis virus (IBV) [180], or environmental stressors such as elevated ammonia levels [181]. Both types of factors can damage the ciliated epithelium of the chicken’s respiratory tract, making it a liable entry for APEC [32,33]. Maintaining good ventilation, regular change of the litter, consistent removal of the debris, and adjusting the bird density can help keep the ammonia level inside the poultry house low and consequently protect the integrity of the bird’s respiratory epithelium [182]. Vaccinating the birds against viral diseases such as IBV can protect the bird against the primary viral infections and lower the probability of any secondary bacterial infections, including APEC. In addition, proper nutrition enhances the bird’s overall health, boosts immunity, potentiates the resistance of the bird against infection, and improves the clearance of the pathogen from the body [183].

APEC is a major cause of early chick mortality, often resulting from vertical transmission of the pathogen to chicken embryos upon oviduct infection or via contaminated eggshell. Several measures can be implemented to prevent both routes of the vertical transmission of APEC, such as raising breeds that are genetically more immune to APEC or ensuring high eggshell quality [28,31]. Good eggshell hygiene could be maintained by minimizing the percentage of floor eggs, which are more prone to contamination, as well as through proper cleaning and disinfection of the eggs and general decontamination and sanitization of the poultry house [28,31]. Establishing a proper biosafety system to restrict the unnecessary entry of vehicles and workers to the poultry house, besides the control of rodents, insects, and wild birds, can further reduce the risk of contaminating the poultry premises [23,29].

### 6.2. Antibiotics

Antibiotics form an essential control strategy for bacterial infections in the livestock [184]. They are used to treat a broad spectrum of both Gram-positive and Gram-negative bacteria, including APEC. Antibiotics can be applied in feed or drinking water or through parenteral administration—such as subcutaneous or intramuscular—for larger animals [185]. Several antimicrobial agents from various classes have been used to treat and control avian colibacillosis. These include aminoglycosides (e.g., gentamycin and neomycin), penicillins (ampicillin and amoxicillin), tetracyclines, sulfonamides, quinolones (e.g., enrofloxacin), cephalosporins (e.g., ceftiofur), phenicols (e.g., chloramphenicol), lincosamides (e.g., lincomycin), macrolides (e.g., erythromycin), and polymexin (e.g., colistin) [31,186]. Previous studies discussed the efficacy of some of these antibiotics for curing APEC and suggested quinolones (e.g., enrofloxacin and norofloxacin), tetracyclines (e.g., oxytetracycline and doxycycline), and sulfonamides (e.g., sulfadimethoxin) as effective candidates for controlling the disease [187,188,189,190]. However, the uncontrolled use of antibiotics in animal production has resulted in a mounting resistance to antimicrobials within microbial communities [191]. On poultry farms antibiotics are extensively used as a prophylactic measure to prevent infections and promote growth despite the legislation to reduce the use of the antimicrobial growth promoters (AGPs) [192,193,194]. This, in turn, has contributed to the spread of resistance on poultry farms [195]. APEC strains with multidrug resistant profiles were frequently reported in the cases of avian colibacillosis [80,161,196,197,198].

The resistance observed in APEC is against multiple antibiotics such as penicillin, cephalosporins, tetracyclines, quinolones and aminoglycosides [199,200]. The multidrug resistant phenotype of APEC is indicative of a genome rich in resistance determinants. For instance, genes conferring the resistance to quinolones (e.g., *qnrA*, *qnrB*, and *qnrS*), tetracycline (e.g., *tetA* and *tetB*), sulfonamides (e.g., *sul1* and *sul2*), aminoglycosides (e.g., *aadA*), and the β-Lactam antibiotics (e.g., blaTEM) are prevalent in *E. coli* isolated from both healthy and diseased chickens [31,152,201,202,203,204]. Importantly, antibiotic resistance genes are usually carried on mobile genetic elements (MGEs) such as plasmids, insertion sequences, and transposons [205,206]. The association of antibiotic resistance with various plasmid replicons such as incP, incI1, incA/C, and incFI has been previously reported in APEC [207,208]. IncF plasmids such as FIA, FIB, and FIC were previously linked to quinolone resistance in APEC [209]. Furthermore, the class 1 and 2 integrons have been associated with multiple antibiotic resistance genes such as trimethoprim (*dfrA*), streptomycin (*aadA*), and erythromycin (*ereA*), aminoglycosides, and sulfonamides in *E. coli* isolated from healthy and diseased birds [210,211,212,213].

The association between antimicrobial resistance and MGEs promotes the horizontal spread of the resistance among bacteria [214,215]. It also could exacerbate the infection in case resistance genes and virulence determinants were genetically linked, leading to the potential spread of both resistant and virulent strains [117,216,217]. Hence, the daunting antimicrobial resistance in poultry production imposes a serious challenge to the antibiotics as a control strategy of the disease on poultry farms by drastically decreasing the efficacy of the antibiotics, leading not only to the failure of the treatment but also the selection for resistant pathogens within the microbial populations [216,218,219]. This provides the drive toward alternative and innovative solutions.

### 6.3. Phytochemicals

Phytochemicals, also known as phytobiotics, are bioactive compounds extracted from plants and supplied in animal feeds to enhance productivity [220]. Due to their antimicrobial activity, they have been suggested as promising alternatives to antibiotics for tackling antibiotic-resistant bacteria [221,222]. Around 1340 plant species with known antimicrobial activity are available [223]. Various antimicrobial compounds from plants such as thyme, chicory, coriander, aloe vera, and turmeric have been incorporated into production of livestock including poultry, pigs, and ruminants as alternatives to antibiotics and for promoting growth [224,225]. Phytochemicals are classified into various categories based on their chemical structures and properties, including alkaloids, flavonoids, phenolic compounds, saponins, and essential oils (EOs) [226]. Extensive efforts have been made to isolate and purify these bioactive compounds to enable their proper identification and application [227]. They exert their antimicrobial effects through various mechanisms, including inhibition of DNA supercoiling, interference with protein synthesis and efflux pump activity, disruption of bacterial cell membranes, and interaction with key bacterial enzymes [222].

Phytochemicals have been used frequently for the treatment of multiple avian pathogens such as APEC [228,229], *Salmonella* [230], *Campylobacter* [231], *Clostridia* [232], *Staphylococcus aureus* [233,234], and Coccidia [235]. Despite their benefits in controlling pathogens and promoting animal growth, the use of phytochemicals in animal production faces several critical challenges. One major hurdle is the poor understanding of their pharmacokinetics, bioavailability, and biotransformation, which complicates their usage and commercialization [236,237,238]. Other limitations relate to animal performance; for instance, when administered at high concentrations, some phytochemicals may exert an anti-nutritional effect represented by decreasing the digestion of certain nutrients and limiting the availability and absorption of some trace elements like zinc and iron [239,240,241]. Furthermore, some EOs can adversely affect the palatability of the animal ration, leading to reducing feed intake and lowering the performance [242]. Also, the use of EOs can be economically burdensome due to the difficulty in determining their effective dosage in animal feed, largely attributable to their highly volatile nature [243,244], and their high minimum inhibitory concentration (MIC), which decreases their cost-effectiveness [245]. The potential toxicity of phytochemicals should be considered carefully [246,247,248], and a precise qualitative and quantitative analysis of the plants and their bioactive compounds is a must to ensure their safety before use [249]. However, the complex composition of phytochemicals poses some difficulties for the systematic evaluation of their safety and efficacy [245].

### 6.4. Vaccines

Vaccines have always been a very effective immunological means of controlling microbial infections throughout the animal production process [250]. In poultry, various kinds of vaccines, including killed, live-attenuated, and subunit vaccines, are available for controlling bacterial and viral diseases [251]. The live-attenuated vaccines are based on introducing well-characterized mutations into the genome of the wildtype strain, rendering it unfit for initiating an infection [252,253]. On the other hand, the subunit vaccine uses a specific part of the pathogen, typically a certain purified antigen, which is delivered via various carriers such as liposomes [254]. Delivering this antigenic molecule via another attenuated bacterium is referred to as recombinant vaccine technology [254].

Continuous efforts have been ongoing to find a good vaccine candidate for controlling avian colibacillosis [255,256,257,258,259,260,261]. Table 1 summarizes some of the experimental attempts of testing several types of potential APEC vaccines in poultry.

A smart technology that has been tested for immunizing chicken against colibacillosis is the bacterial ghost vaccine. This type of vaccine is based on emptying the content of the bacterial cell through a pore created in the cell membrane. This process is accomplished through the controlled expression of the lysis gene E of the phage φX174 and retaining the cell membrane intact with its surface antigens [262,263]. As a result, ghost vaccines combine the advantages of strong immunogenicity because they keep the membrane-associated epitopes and safety because they are unable to replicate [263]. Either an APEC or a non-APEC strain (e.g., lab strain) exogenously expressing specific APEC proteins can be used for ghost vaccine preparation [264,265]. Although most vaccine types share the advantage of being highly immunogenic and able to stimulate humoral immunity, important challenges need to be addressed when they are used. These challenges could be summarized in three key aspects: (a) protection, (b) safety, and (c) applicability. Regarding protection, a major limitation that faces most potential vaccine candidates is their failure to confer cross-protection against heterologous APEC strains or serotypes [256,259,266]. Therefore, autogenous vaccines prepared from the respective field strains may be more effective; however, they impose restrictions on the vaccine’s applicability and production process [259,267,268].

Despite some previous studies reporting either failed or weak protection in the vaccinated birds [269,270,271], successful vaccines develop immunity in a few-week period of time during which the birds are liable to infection especially during the first few days after hatching [260,261,272]. This, in turn, underscores the importance of the passive immunity transferred from the breeder hens to the hatchlings in protecting chicks during their early life [273]. However, failure of maternal immunity transfer to the offspring or the inability to protect the newly hatched chicks by the vertically transmitted antibodies may occur [260,261,268,273]. The safety of the vaccine exemplified by the reversion of the vaccine avirulent or live-attenuated strain to the virulent phenotype and the side effects from large vaccination doses must be considered carefully [255,256,274,275]. In terms of vaccine applicability, mass vaccination of the entire flock requires an easy administration route, such as spray or in drinking water. However, some vaccines could be more effective when administered through a method that is not very practical for mass vaccination programs such as injection or better still in ovo inoculation [265]. Furthermore, many vaccines may require a booster dose to elicit a strong immune response, which imposes a financial burden on the vaccination program.

Finally, despite the numerous attempts to develop a potent avian colibacillosis vaccine, only a few candidates have reached the market and become commercially available, such as the ∆*aroA* live-attenuated vaccine (Poulvac^®^ *E. coli*, Zoetis, U.S.) [276], the inactivated vaccine (Nobilis *E. coli* Inac., MSD Animal health, U.S.) [277], and the Δ*crp* live-attenuated vaccine (Gall N tect CBL, Nisseiken Co., Ltd., Tokyo, Japan) [278]. These vaccines are based on the APEC O78 serotype, and although they have been proven to be effective and safe, they provide weak immunity against heterologous serotypes. However, the most studied commercial vaccine, i.e., the ∆*aroA* Poulvac vaccine, showed variable results regarding cross-protection [279,280,281,282]. It is worth noting that the failure to protect offspring via passive immunity from parents vaccinated with Nobilis or Poulvac vaccines has been previously reported [268,283]. Collectively, the shortage of potent commercial vaccines against avian colibacillosis and the inefficient cross-protection against APEC serotypes other than the progenitor strains of the vaccines represent the primary challenges that face APEC control via vaccination.

**Table 1:** Vaccine trials to control APEC.

**Inactivated/Killed vaccine**				
**Vaccine strain serotype**	Inactivation	Vaccination route	Bird	Reference
**APEC** **(strain KAI-2/O78)**	Formalin inactivated	Eye drop and coarse spray	Chicken	[257]
**Monovalent** **(*E. coli* O78 or O1)**	Formalin inactivated	Subcutaneous	Chicken	[267]
**Multivalent** **(O18, O78 and O111)**	Unclear	Intramuscular	Chicken	[259]
**O157**	Formalin inactivated	Subcutaneous	Chicken	[272]
**Bivalent** **(*E. coli* 19-381 and 19-383-M1)**	Formaldehyde inactivated	Intramuscular	Chicken	[273]
**Live-attenuated vaccine**				
**Vaccine strain serotype**	Mutation	Vaccination route	Bird	Reference
**O2**	*carAB* operon mutation	Oral	Turkeys	[284]
**O78 and O2**	∆*cya* and ∆*cya*∆*crp*	-		[255]
**O78** **O2**	∆*cya*∆*crp* ∆*cya*∆*crp*	Spray	Chicken	[269]
**O78**	∆*galE*, ∆*purA*, and ∆*aroA* (single mutation)	Spray	Chicken	[256]
**O78**	∆*aroA*	Spray and oral	Chicken	[266]
**O78**	*Crp* deletion	Spray, eye drop, and in ovo	Chicken	[278]
**Subunit vaccine**				
**Strain serotype**	Antigen	Route	Bird	reference
**O1**	Pili protein	Subcutaneous	Chicken	[285]
**O1, O2, and O78**	Pili proteins of the three serotypes (Multivalent)	Subcutaneous	Chicken	[286]
**Unclear**	Sugar-binding domain of FimH (FimH156)	Intramuscular or intranasal	Chicken	[270]
**Unclear**	Sugar-binding domain of PapGII (PapGII196)	Intramuscular	Chicken	[271]
**Unclear**	Iss protein fused to glutathione S-transferase (GST-iss)	Subcutaneous	Chicken	[287]
**Unclear**	Liposome-encapsulated mixture of rough LPSs of core types R1, R2, R3 and R4.	Intramuscular	Chicken	[288]
**Unclear**	Iss protein, fused to glutathione S-transferase (GST-iss)	intramuscular	Chicken	[274]
**APEC O78 and NEMC O18**	Recombinant antigens (rAg) including (EtsC, OmpA, OmpT, and TraT)	Subcutaneous	Chicken	[48]
**Unclear**	Enterobactin	Subcutaneous	Chicken	[260,261]
**Recombinant vaccine**				
**Recombinant strain**	Exogenous genes of *E. coli*	Route	Bird	Reference
**∆*lon* ∆*cpxR* ∆*asdA*16 *S.* Typhimurium (JOL912)**	P-fimbriae (*papA* and *papG*) Aerobactin receptor (*iutA*) CS31A surface antigen (*clpG*)	Orally	Chicken	[275]
***S.* Typhimurium, ∆*cya*-27, ∆*crp*-27,** **∆asdA16**	*Ecp* operon encoding *E. coli* pilus	Orally	Chicken	[289]
***Lactobacillus saerimneri* M-11**	Fimbrial subunit A (*fimA*) Outer-membrane protein C (*ompC*)	Orally	Chicken	[290]

### 6.5. Bacteriophages

Bacteriophages (commonly referred to as phages or “bacteria eaters”) are viruses that specifically infect and lyse bacterial cells [291]. They were early recognized as a potential bactericidal tool for curing bacterial infections [292]. A bacteriophage could be either a lytic or lysogenic type. A lytic or virulent bacteriophage injects its genetic material into the bacterial cell, hijacks the cell, and replicates until the cell finally ruptures releasing progeny [293,294]. Conversely, a lysogenic or temperate bacteriophage integrates its genetic material into the bacterial chromosome and replicates concurrently with the bacterial genome without any lytic activity on the bacterial cell [293,294]. Due to their bactericidal nature, lytic bacteriophages are suitable as an antimicrobial solution for bacterial control [295], and have been applied for curing bacterial infections in humans [296,297], farm animals [298], aquaculture [299], and plants [300]. In poultry production, several trials have been conducted using bacteriophage for the control of significant poultry pathogens such as *Salmonella* and *Campylobacter* [301]. In most trials, one bacteriophage or a mixture of different bacteriophages (i.e., phage cocktail) was administered orally, resulting in a significant reduction in the viable counts of the target bacteria [301]. Considering avian colibacillosis, bacteriophage administration to chicks via different routes (i.e., intramuscular and intratracheal) resulted in reduction of various serotypes (i.e., O1, O2 and O78) of APEC [302,303,304]. The application of a phage cocktail has also showed successful control of APEC in a more efficient way than single-phage treatments [304]. Furthermore, applying bacteriophages to the litter or via spraying on hatching eggs could be used as a prophylactic measure to prevent colibacillosis as it helped reduce the mortality rate and enhance the body weight of the broiler chicks [305].

Bacteriophage therapy presents severe limitations: first, the high specificity of bacteriophage adsorption to bacteria, which results in targeting only one single species or a subset of susceptible bacterial strains within a single species [291,306]. As a consequence, precise diagnosis of the causative bacterial agent of the infection and its susceptibility is essential to selecting a suitable bacteriophage or bacteriophage cocktail, although even this is not a guarantee for efficiency [291,307]. A second important limitation is the rapid development of phage resistance in bacteria [308,309]. Mutations that block or alter the bacteriophage receptor, preventing the adsorption process and bacteriophage DNA restriction, which cuts and inactivates the bacteriophage genome, are two resistance mechanisms that render bacteriophage treatment ineffective [310,311]. A further consideration is the role of bacteriophages in transduction and genome reassortment that could contribute to the spread of antimicrobial resistance and virulence among bacterial strains in the animal [312,313,314].

### 6.6. Probiotics

Probiotics, or diet-fed microbials (DFMs), are non-pathogenic bacteria that, when administered to the animal host, exert a beneficial effect on its health [315]. Various bacterial species such as *Bifidobacterium* spp., *Streptococcus* spp., *Bacillus* spp., *Lactococcus* spp., and *Lactobacillus* spp., as well as yeasts, have been exploited as probiotics for animal production [316]. Probiotics are claimed to offer multiple benefits in farmed animal production, such as enhancing growth performance, improving host immunity, modulating the gut microbiome, and controlling pathogenic bacteria [317]. For poultry, birds that received DFM such as *Bacillus subtilis* or *Lactobacillus* spp. showed an improved immune response, represented by enhanced IgA secretion into the intestine and modulating intraepithelial lymphocytes (e.g., CD4 cells) [318]. Probiotic administration to newly hatched chicks introduces beneficial changes to the gut microbiome by promoting beneficial taxa and limiting harmful ones in the bird’s gut [319]. These diet-fed beneficial bacteria can compete with pathogenic strains, preventing them from colonizing the epithelial surfaces through competitive exclusion [320,321]. The inclusion of *Bacillus subtilis* or *Lactobacillus*-based probiotics in poultry feed has been shown to reduce the incidence of some bacterial pathogens such as *Salmonella* spp. and *Clostridium perfringens* [322,323,324]. Regarding APEC control, the probiotic candidate *Enterococcus faecalis*-1, administered to broiler chicken in drinking water, enhanced the immune response, improved growth, and reduced invasion by APEC O78 in the challenged birds [325]. Another probiotic mix containing *Bacillus subtilis* MORI 91, *Clostridium butyricum* M7, and *Lactobacillus plantarum* K34 also demonstrated a protective effect in the birds challenged with APEC O78 [326]. The administration of commercially available probiotics, such as Aviguard and Gro2MAX, reduced transmission and excretion of extended-spectrum β-lactamase (ESBL)-producing *E. coli* in treated chicks [327,328,329].

A critical challenge of probiotics usage is their potential to acquire and disseminate antimicrobial resistance [330]. Despite their avirulent genotype, strains of the probiotic species *Enterococcus faecium*, isolated from various commercial products intended for animal usage in the US, exhibited a multidrug-resistant phenotype to medically important antibiotics [331]. This consequently highlights the potential hazards associated with the probiotics in case successful transfer of resistance from a probiotic strain to a pathogenic one took place rendering them difficult to treat. Notably, balancing the use of both probiotics and antibiotics is critical, as the administration of enrofloxacin following the commercial probiotic Aviguard was shown to negate its beneficial effect and restore the enrofloxacin-resistant *E. coli* population in the treated chickens [332]. The safety of probiotics is also critical. The potential of a probiotic to induce infection, allergic reaction, or adverse metabolic effect is a significant risk that must be pondered carefully [330]. A study conducted in China to assess the safety of animal-use commercially available probiotics found that one-third of the 92 products tested were contaminated with life-threatening pathogens such as *Klebsiella pneumoniae*, and one of them contained an anthrax toxin-positive *Bacillus cereus* strain [333]. Hence, ensuring the safety of the proper probiotic strain is crucial [320,334]. There are many claims made for the efficacy of probiotics and, whilst in some cases there is evidence for marginal reduction of gastrointestinal pathogens, their antimicrobial effects are largely strain-specific, and the underlying mechanisms of action remain poorly understood [335].

### 6.7. Prospective APEC Control

#### 6.7.1. Antimicrobial Nanoparticles

Nanoparticles are extremely small, with three nanometer-scale (1–100 nm) dimensions, and exhibit physical, chemical, and biological properties distinct from their bulk counterparts [336]. Based on their source, nanoparticles can be classified into two main types: organic and inorganic. Organic nanoparticles, such as ferritin and liposomes, come from carbon-based sources. In contrast, inorganic nanoparticles do not contain carbon such as metal-based and metal oxide-based nanoparticles [337]. Many metals such as aluminum, gold, silver, lead, copper, and iron are involved in the synthesis of both metal and metal oxide nanoparticles. The method used for producing nanoparticles varies depending on their type. However, in general, two main approaches are commonly considered: (a) the top-down approach and (b) the bottom-up approach [338]. In the top-down approach, nanoparticles are produced by breaking down bulk material into smaller particles until the desired nanoscale size is achieved. In contrast, the bottom-up approach involves building up the nanoparticles by assembling simpler atomic or molecular building blocks [339].

Either as vehicles for antibiotic delivery or as antimicrobial agents themselves, nanoparticles stand out as a promising solution in the fight against multidrug resistant bacteria [340]. Nanoparticles as drug carriers help overcome the pharmacokinetic/dynamic hurdles that could affect the efficacy of the antibiotic. For instance, nanocarriers could potentiate the ability of an antibiotic to cross biological barriers such as the blood–brain barrier and cell membranes, allowing the antibiotic to reach resistant intracellular infections such as *Salmonella*, *Listeria*, and *Brucella* [341]. The minute size of nanoparticles allows their efficient entry to the target cell via phagocytosis and endocytosis [342]. In addition, nanoparticles such as liposomes and polymeric nanoparticles improve the bioavailability of the drug by enhancing its solubility and prolonging its systemic circulation to achieve the maximum effect [343]. They also protect the antibiotic against enzymatic degradation, which enhances the efficacy of the antibiotic and reduces the dose required to exert an effect [344]. The aforementioned advantages encouraged scientists to use antibiotic-loaded nanoparticles for the treatment of many infectious pathogens such as *E. coli*, *Pseudomonas*, *Mycobacterium tuberculosis*, and *Staphylococcus aureus* [345].

Interestingly, nanoparticles could also be applied as antimicrobials on their own without requiring antibiotics to be conjugated. Generally, they exert their antimicrobial effect either by causing physical damage to cell membrane or changing the cellular chemistry, rendering it toxic to the bacterial cell [346,347]. The physicochemical characteristics of nanoparticles potentiate their role as antimicrobial agents [348]. In terms of physical characteristics, the size and shape of nanoparticles affect their antimicrobial activity. Previous reports discussed the superior antimicrobial activity of small, spherical nanoparticles compared to the triangular or rod-shaped ones [349,350]. The enhanced antimicrobial effect of these nanospheres is likely due to their increased surface area compared to other shapes, which fortifies their reactivity, release of toxic ions, attaching to bacterial cell membrane, penetration of the cell, and damaging of the DNA [349,350]. Furthermore, others reported that anisotropic nanoparticles with sharp edges are more able to penetrate the bacterial cell membrane, leading to cell rupture and release of content, and thus have a stronger bactericidal activity than nanoparticles with rounded edges [351]. Surface charge is another key factor influencing antimicrobial activity. Positively charged nanoparticles are particularly effective against Gram-negative bacteria, as they readily interact with the negatively charged bacterial membranes, leading to membrane disruption and bacterial death [352,353]. Additionally, the chemical composition of nanoparticles contributes to their bactericidal effect. Metal-based nanoparticles can actively release toxic metal ions within bacterial cells [353]. Once inside, they also generate reactive oxygen species (ROS), such as hydrogen peroxide, superoxide anions, and hydroxyl radicals. These ROS, along with the released metal ions, cause DNA damage and disrupt essential enzymatic functions, ultimately leading to bacterial cell death [347,354,355]. Metal and metal oxide nanoparticles have been extensively studied for their effectiveness against a wide range of resistant pathogens, including Gram-negative bacteria such as *E. coli*, *Salmonella*, *Klebsiella*, and *Campylobacter* [356,357,358,359,360], as well as Gram-positive bacteria, such as *Staphylococcus aureus* and *Clostridium* spp. [361,362,363]. The metal nanoparticles are effective individually; however, combinations of more than one type of metal nanoparticles exhibited an enhanced antimicrobial effect due to the synergism between them [364,365].

Although nanoparticles provide a promising solution as antimicrobials and antibiotic delivery vehicles, challenges that might delay the progress in this field need to be fully understood. As living organisms with adaptive capabilities, bacteria have managed to develop resistance against some antimicrobial nanoparticles [366]. Several mechanisms have been discussed that help the bacteria to resist the antimicrobial effect of nanoparticles, primarily by decreasing the entry or increasing the exit of the nanoparticles in and out of the bacterial cell. Reducing the numbers of porin molecules on the bacterial surface, especially OmpF, is one strategy adopted by bacteria to decrease the entry of small nanoparticles into the cell [367]. Furthermore, overexpression of the flagellin protein forms a matrix around the bacterial cell that aggregates the nanoparticles and prevents their contact with the cell membrane [367]. Sublethal doses of metal and metal oxide nanoparticles can stimulate the upregulation of efflux pumps which expel the metal and metal oxide ions out of the bacterial cell before killing it [368]. Therefore, ongoing research is essential to deepen our understanding of mechanisms of bacterial resistance to nanoparticles, which is critical for developing effective strategies to overcome this challenge. Conjugating the NPs with natural extracts could help suppress the mechanisms of bacterial resistance to NPs, thereby restoring their antimicrobial efficacy. For example, the pomegranate rind extract has been shown to suppress bacterial flagellin and restore the bacterial sensitivity to silver NPs [366].

Another significant challenge in applying nanoparticles as antimicrobials is their potential toxicity to non-target organisms, including beneficial microbiota and eukaryotic host cells. The mechanisms that make NPs effective against pathogens—such as ROS generation—may also harm other biological systems. For example, ROS produced by metal-based NPs can induce oxidative stress in eukaryotic cells, leading to DNA damage and destruction of organelles [369,370,371]. In vivo studies have shown that oral administration of NPs in mice can disrupt gut microbiota, causing dysbiosis, promoting the growth of opportunistic pathogens, and triggering intestinal inflammation [372,373]. These findings highlight the need for comprehensive safety assessment of NPs. With the growing production and widespread application of NPs, their potential environmental burden warrants serious consideration. Nanoparticles can enter the environment at various stages of their life cycle—during production, usage, and disposal—leading to contamination of soil and water [374]. This highlights the need for a comprehensive understanding of their environmental fate, impact, and the development of standardized methods for their detection and quantification. Once released, nanoparticles may persist in the environment or undergo various transformations, such as sulfidation, aggregation, sedimentation, dissolution, and adsorption, all of which can significantly influence their fate and toxicity [375,376]. While notable progress has been made in the risk assessment of nanoparticles, further research is essential to unravel the mechanisms of their toxicity in both humans and animals, as well as their interactions with environmental matrices. Such understanding is vital for ensuring the safe and sustainable application of these promising antimicrobial agents.

#### 6.7.2. Enzybiotics (Endolysins)

Enzybiotics are enzymes capable of killing bacteria and thus act as antibiotics. They are primarily phage-encoded enzymes, used by bacteriophages during the late stage of their lytic cycle to lyse the bacterial cell and release the newly formed progeny [377,378]. The model enzybiotics are endolysins that act by digesting the peptidoglycan layer of the bacterial cell wall [379]. During the phage infection cycle, phage particles enter the bacterial cell, replicate, and ultimately burst the cell releasing more phage particles to infect neighboring bacterial cells. Endolysins play a crucial role in the final stage by intensely degrading the peptidoglycan layer, thereby perforating the cell wall resulting in osmotic rupture [380]. There are two different types of endolysins to adapt targeting different types of bacterial membranes: Gram-negative-targeting endolysins and Gram-positive-targeting endolysins [381]. The Gram positive-targeting endolysins typically consist of two domains connected together by a flexible linker. One domain at the C-terminal of the polypeptide chain acting as a cell wall binding domain (CBD), and the other at the N-terminal which is the catalytic domain or the enzymatic activity domain (EAD) [382,383]. On the other hand, most Gram negative-targeting endolysins often comprise only one globular catalytic domain [382,383]. Endolysins act on the peptidoglycan layer of the bacterial cell wall via different mechanisms, depending on the activity of their EAD domain. They can function as glycosidases, amidases, or endopeptidases—respectively breaking the glycosidic bond between residues of the glycan chain, the amide bond between the glycan chain and the peptide side chain, or the peptide bond between adjacent peptide side chains [384,385]. Thus, they have been exploited as promising next-generation antibiotics in the fight against the antimicrobial resistance [386]. A plethora of studies have demonstrated the potency of bacteriophage endolysins against a broad spectrum of Gram-positive and Gram-negative bacteria affecting both animals and humans [387]. Among Gram-positive bacteria, *Staphylococcus aureus*, *Bacillus anthracis*, and *Streptococcus* spp., such as *S. agalactia*, *S. pneumoniae*, and *S. suis* have shown susceptibility to endolysin-based treatments in animal models [388]. The efficacy of the endolysins against these pathogens was represented by lowering bacterial loads and improving survival rates of the treated animals [388]. Moreover, several Gram-negative bacteria such as *Escherichia coli*, *Klebsiella pneumonia*, and *Pseudomonas aeruginosa* were also susceptible to the antimicrobial activity of endolysins [389].

Endolysins, as antimicrobials, are considered superior to bacteriophages in terms of both activity spectrum and speed of action [390]. However, the most significant advantage of endolysins over bacteriophages is the lower probability of bacteria to develop resistance to endolysins than to bacteriophages [390,391,392]. This advantage is likely attributed to the cell wall binding domain (CBD) of endolysins, which specifically recognizes and binds to highly conserved and essential epitopes within the bacterial cell wall. Mutations in these regions are of low chance, as they would compromise bacterial viability [393,394]. This prevented the bacteria cultured on agar plates with low concentrations of endolysins from developing resistant clones even after several passages [393]. Therefore, the low propensity for resistance development makes endolysins particularly promising antimicrobials compared to bacteriophages and conventional antibiotics in the fight against resistant pathogens. Despite their advantages, endolysins have certain limitations. Due to their peptidoglycan-targeting mode of action, their bactericidal efficacy depends on the structural characteristics of the target bacteria. In the case of Gram-positive bacteria, the bacterial interface with the environment is an outer peptidoglycan layer, readily accessible to endolysins, rendering these bacteria easy targets for lysis. On the other hand, Gram-negative bacteria have an outer glycolipid membrane that hinders the accessibility of endolysins to the underlying peptidoglycan layer, thereby reducing their efficacy [395,396]. Consequently, only a limited number of endolysins with an intrinsic ability to lyse Gram-negative bacteria are available [389,396,397]. This presents a challenge either to find new endolysins that are naturally effective against Gram-negative bacteria or to develop strategies to make endolysins more penetrative.

One approach is combining endolysins with a membrane permeabilizer such as EDTA [398]. However, in some cases, the endolysin could counteract the effect of the permeabilizer as with Vibrio parahaemolyticus, where the bacterium used the proteinaceous endolysin as a nutritive substrate to boost its growth after the effect of the permeabilizer [399]. Another strategy to overcome the limitation in Gram-negative bacterial targeting by endolysins is via molecular engineering of the wildtype enzymes “Artilysins” [400,401]. For example, fusing endolysins with permeabilizer moieties, such as the polycationic nonapeptide (PCNP) or the sensitizer peptide KL-L9P, can disrupt the outer membrane of Gram-negative bacteria. This allows the endolysins to reach the peptidoglycan layer, enhancing their ability to access and lyse the bacterial cell [402,403]. These engineered endolysins showed improved efficacies against multiple Gram-negative bacteria despite the extra effort required for their engineering [404]. Moreover, the continuous in vitro evolution of endolysins via molecular engineering produced enzymes that can target intracellular pathogens [405,406].

Despite the efficiency of such engineered endolysins, they raise a concern about the eukaryotic cell interaction potential, i.e., cell toxicity. In addition, being of a proteinaceous nature, the immunogenicity and anaphylaxis potential of endolysins are always questionable, especially when seeking the control of systemic infections that require administration of the enzymes in the circulation. Some preclinical studies assured the safety of endolysins in terms of general toxicity and allergenicity [407,408,409]. However, the expanding arsenal of endolysins necessitates further studies to ensure their safety. Another aspect of the antimicrobial endolysins’ application is their short half-life in the circulation ranging from minutes to hours due to lysosomal degradation and kidney excretion [410]. This short half-life imposes further challenges to both application and production of endolysins, as it requires booster dosing to achieve an effect as well as high concentrations of the protein in the used formulation. Consequently, this increases the chance of side effects of multiple doses, reduces the stability of the protein in the formulation, and reduces cost-effectiveness [383,395,410]. Extensive research has been conducted to improve the characteristics of endolysins and prolong their serum half-life with promising results [411,412].

Although endolysins constitute a very promising solution to control the dilemma of antimicrobial resistance, a few products have reached the market—mainly as feed additives for veterinary use, such as FORC3 and Axitan. This limited commercialization could be attributed to several unresolved challenges, including difficulties in large-scale production, formulation stability, delivery methods, and the complex regulatory pathways for approval of biologically derived antimicrobials. Addressing these hurdles is critical to translate laboratory successes into clinically and commercially viable therapies.

#### 6.7.3. CRISPR-Based Antimicrobials

Bacteria have evolved different defense mechanisms to protect themselves against mobile genetic elements from the surrounding environment especially bacteriophages. The most recently described system of defense is CRISPR (Clustered Regularly Interspaced Short Palindromic Repeats), an adaptive immune system in prokaryotes used to inactivate foreign genetic material that enters the bacterial cell [413,414]. CRISPR is an array of short, repeated DNA segments called CRISPR repeats separated from each other at regular intervals by variable sequences called spacers. Such arrays have been identified in the genomes of most archaea and nearly half of the known bacterial species [415,416]. The CRISPR-defense response consists of three key stages: (A) adaptation of the system by incorporating pieces from the invading genetic material known as spacers into the CRISPR array, effectively updating its memory of past threats; (B) expression of the CRISPR array and processing the transcripts into mature RNA guides; and (C) interference, where the mature RNAs guide the CRISPR-associated proteins to complementary regions in the foreign genetic material (known as protospacers), where they bind and neutralize the invader [417,418].

**Figure 2:**
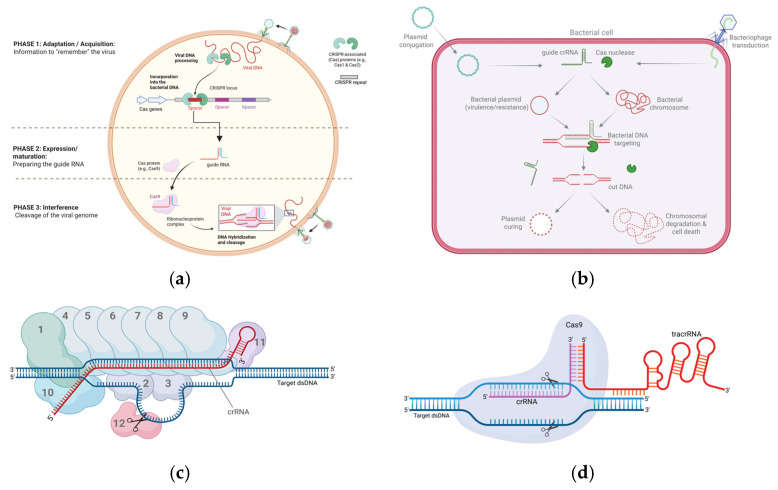
Representation of CRISPR mechanism of action and CRISPR-Cas classes I and II. (**a**) General CRISPR mechanism of action represented by three main stages: adaptation, expression, and interference. (**b**) CRISPR antimicrobial delivery into target bacteria via conjugation or transduction for either plasmid curing or bactericidal chromosomal targeting. (**c**) Class I of CRISPR-Cas, represented by CRISPR-Cas3 (type I-E). Numbers 1–11 represent components of the cascade complex (1 = CasA; 2, 3 = two units of CasB; 4–9 = 6 units of CasC; 10 = CasD; 11 = CasE). Number 12 represents the effector Cas3 protein which induces a nick in the displaced non-target strand. (**d**) Class II of CRISPR-Cas, represented by CRISPR-Cas9 (type II-A). Only one effector protein is involved in this system, the Cas9 protein. The guide crRNA is partially hybridized to an RNA molecule known as tracrRNA. The Cas9 protein induces double-stranded break in both target and non-target strands.

Differences among CRISPR loci in the composition of the Cas proteins and the effector modules allowed the classification of the CRISPR-Cas systems into two major classes: class 1 and class 2 [419,420]. Class 1 includes the CRISPR systems where the effector complex consists of multiple protein subunits such as the CRISPR types I, III, and IV [420,421]. On the other hand, the CRISPR systems of class 2 are defined by possessing a single-unit effector protein such as the CRISPR types II, V, and VI [420,422]. These six CRISPR types are further subdivided into 33 subtypes according to the composition and architecture of the Cas operon, as well as the Cas1 protein phylogeny [420,423]. Figure 2 illustrates the general CRISPR mechanism of action and a representative CRISPR type of each class.

Both the expression and interference stages constitute the main pillars for the CRISPR-based antimicrobials. Delivering a CRISPR array that includes a self-targeting spacer besides the CRISPR-associated genes into the bacteria will target the bacterial own DNA, imposing a toxic stress on it. Bacteria do not tolerate self-targeting by CRISPR systems due to their inefficient DNA repair mechanisms which render chromosomal targeting lethal [424]. A strict requirement for CRISPR antimicrobials is to ensure the presence of a protospacer adjacent motif (PAM) flanking the targeted protospacer. PAM is a few-nucleotide sequence acting as a signal for Cas proteins to cut the genetic material upon the complementary binding between the guide RNA and the protospacer [425]. This criterion must be considered during the spacer selection process to guarantee the efficiency of targeting.

An early trial to prove this concept was conducted by Gomaa et al., who targeted the *E. coli* K-12 by transforming the bacterium with plasmids expressing the CRISPR-Cas proteins of the type I-E CRISPR system (i.e., cascade complex and Cas3) and a minimal CRISPR array containing a single spacer that was modified to target a different chromosomal gene each time [426]. Following this experiment, other trials tested different CRISPR-Cas systems and various delivery strategies. CRISPR-Cas9-based antimicrobials were delivered either via plasmid conjugation [427,428] or phage-based-delivery [429,430] to target different pathogens such as *Salmonella* Typhimurium, *Staphylococcus aureus*, and *Enterococcus faecalis*. These trials and many others have proven the efficiency of different CRISPR-Cas systems to target several bacterial pathogens [431]. Furthermore, a key advantage of CRISPR technology is its high specificity, through which CRISPR-based antimicrobials can efficiently eliminate certain bacterial strains in mixed bacterial cultures, allowing to distinguish between pathogens and commensals [426,429,432,433].

Despite the auspicious future of the CRISPR-based antimicrobials, challenges have been outlined. An important prerequisite for successful application of CRISPR as an antimicrobial is to define a specific target site of the pathogen. Two approaches could be exploited; either a pathogen-focused approach or a gene-focused approach [434]. In the pathogen-focused approach, the target site must be located on the bacterial chromosome to ensure elimination of the pathogen. This target site could either be a virulence gene, a resistance gene, any pathogenicity determinant, or another unique DNA signature of the bacterial pathogen [435,436]. On the other hand, the gene-focused approach ensures the depletion of the gene from the bacterial population rather than the pathogen itself. Therefore, it focuses on targeting plasmid-carried resistance genes, leading to curing of the plasmid, depletion of the gene, and resensitizing of the host bacterium [437,438,439]. It is worth noting that knocking out plasmids that possess toxin/antitoxin addiction systems indicates that targeting plasmids may not only cure the plasmid but can also kill the host bacterium on occasion [440]. Regarding the control of APEC, the target selection process constitutes a major limitation. The precise identification of the APEC pathotype determinants remains vague, with variable combinations of virulence genes used as an indicative of the APEC genotype. Additionally, many virulence determinants are harbored on large virulence plasmids in the bacterium [166,441], which, as discussed before, may set a barrier against using them as targets. However, it is of interest that curing these large virulence-encoding plasmids may render the host bacterium either less pathogenic or even avirulent.

Also, CRISPR delivery remains a crucial challenge to the CRISPR-antimicrobial technology and a critical prerequisite besides target selection. Bacterial conjugation stands as a handy strategy to deliver DNA cargos in the lab environment. However, the efficiency of conjugation to achieve high transfer rates of the plasmids in complex microbial communities is questionable [442,443]. Cell-to-cell contact enhances conjugation efficiency, which might be challenging especially under in vivo conditions [444]. Continuous effort has been exerted either for the hunt for new highly transferable systems or engineering the present ones for improvement [445,446]. Bacteriophages are frequently in use for delivery; however, engineering bacterial plasmids with the required DNA is possibly easier than engineering the genomes of bacteriophages [447]. Furthermore, host range restrictions constitute a critical hindrance against the bacteriophage usage when compared to some conjugative plasmids such as RP4 that are promiscuous with a broad host range [448,449]. However, the limited host range of bacteriophages could be tackled by engineering the tail fiber protein [450,451] or producing cocktails of CRISPR-weaponized bacteriophages; either solution, however, remains a laborious process compared to plasmid manipulation. Despite the high specificity of CRISPR, an off-target effect has been reported, attributed to the variable tolerance of CRISPR systems to the guide RNA–protospacer mismatches [452,453]. Although off-target activity is generally low [454], it can still induce dysbiosis by inadvertently targeting beneficial microbial species [455]. This could be mitigated by utilizing bioinformatics tools to design highly specific guide RNAs [456], modifying the guide RNA through extension or truncation [457], and using engineered Cas9 variants with improved fidelity, such as *Streptococcus pyogenes* Cas9 high-fidelity variant 1 [SpCas9-HF1] [458].

It is worth considering the bacterial resistance to the CRISPR-antimicrobials [459]. Bacteria can resist CRISPR targeting via different strategies, including the inactivation of the CRISPR machinery either by anti-CRISPR proteins (Acr proteins) [460,461], or by mutating the CRISPR array via spacer deletion [426,462,463] or mutating the Cas proteins [459,464]. However, tackling CRISPR resistance is still easier compared to tackling the antibiotic resistance due to the high modularity of the CRISPR-based antimicrobials. Improving the effector Cas protein expression (e.g., Cas9) or using other efficient Cas protein types such as Cas13a or Cas3 could help overcome the resistance due to protein inactivation [433,459,465]. Also, the deleted spacer phenotype can be avoided by preventing recombination of the repeats flanking the spacer either by using the single-guide RNA version of the array or by the incorporation of a terminal degenerate repeat [431,466]. Mutating the target gene to escape killing was also reported as an escape strategy [463,467], which can be solved by multiplexing the CRISPR array to simultaneously target multiple sites [431]. Despite the previous challenges, CRISPR modularity and specificity are still two very important advantages that strongly suggest CRISPR-antimicrobials as a promising alternative to treat resistant pathogens, especially with the continuous efforts undertaken to improve the technology.

## 7. Conclusions

Colibacillosis is a challenging problem for the poultry industry worldwide. Its systemic nature and widespread lesions result in severe losses to egg and meat production. Studious efforts have been exerted to understand the causative bacterium and predisposing factors, and provide suitable control strategies to minimize its impact on poultry production and public health. Although the available identification schemes are quick and valuable in the diagnosis process, the involvement of WGS technologies is changing our view of the pathogen, its determinants, and its zoonotic potential, and these technologies are yet to be more included in the diagnostic procedures.

Importantly, it is crucial to keep the hunt going for an effective strategy to control APEC, especially with the increasing resistance to the conventional antibiotics and the scarcity of commercial vaccines. New technologies such as endolysins, nanoparticles, and CRISPR endonucleases are worth considering and can provide alternative pathways to control the disease. Finally, a multidisciplinary approach that integrates genomic tools, innovative therapeutics, and improved management practices holds the greatest promise for achieving sustainable control of colibacillosis and safeguarding both poultry health and public safety.

## Data Availability

No new data were created or analyzed in this study.

## References

[1] Denamur E., Clermont O., Bonacorsi S., Gordon D. (2021). The Population Genetics of Pathogenic *Escherichia coli*. Nat. Rev. Microbiol..

[2] Peng Z., Wang X., Huang J., Li B., Tang Y.-W., Hindiyeh M., Liu D., Sails A., Spearman P., Zhang J.-R. (2024). Pathogenic *Escherichia coli*. Molecular Medical Microbiology.

[3] Riley L.W. (2020). Distinguishing Pathovars from Nonpathovars: *Escherichia coli*. Microbiol. Spectr..

[4] Pakbin B., Brück W.M., Rossen J.W.A. (2021). Virulence Factors of Enteric Pathogenic *Escherichia coli*: A Review. Int. J. Mol. Sci..

[5] Logue C.M., Wannemuehler Y., Nicholson B.A., Doetkott C., Barbieri N.L., Nolan L.K. (2017). Comparative Analysis of Phylogenetic Assignment of Human and Avian ExPEC and Fecal Commensal *Escherichia coli* Using the (Previous and Revised) Clermont Phylogenetic Typing Methods and Its Impact on Avian Pathogenic *Escherichia coli* (APEC) Classification. Front. Microbiol..

[6] Sora V.M., Meroni G., Martino P.A., Soggiu A., Bonizzi L., Zecconi A. (2021). Extraintestinal Pathogenic *Escherichia coli*: Virulence Factors and Antibiotic Resistance. Pathogens.

[7] Robins-Browne R.M., Holt K.E., Ingle D.J., Hocking D.M., Yang J., Tauschek M. (2016). Are *Escherichia coli* Pathotypes Still Relevant in the Era of Whole-Genome Sequencing?. Front. Cell. Infect. Microbiol..

[8] Martinez-Medina M. (2021). Special Issue: Pathogenic *Escherichia Coli*: Infections and Therapies. Antibiotics.

[9] Clermont O., Bonacorsi S., Bingen E. (2000). Rapid and Simple Determination of the *Escherichia coli* Phylogenetic Group. Appl. Environ. Microbiol..

[10] Clermont O., Christenson J.K., Denamur E., Gordon D.M. (2013). The Clermont *Escherichia coli* Phylo-Typing Method Revisited: Improvement of Specificity and Detection of New Phylo-Groups. Environ. Microbiol. Rep..

[11] Clermont O., Dixit O.V.A., Vangchhia B., Condamine B., Dion S., Bridier-Nahmias A., Denamur E., Gordon D. (2019). Characterization and Rapid Identification of Phylogroup G in *Escherichia Coli*, a Lineage with High Virulence and Antibiotic Resistance Potential. Environ. Microbiol..

[12] Pires-Dos-Santos T., Bisgaard M., Christensen H. (2013). Genetic Diversity and Virulence Profiles of *Escherichia coli* Causing Salpingitis and Peritonitis in Broiler Breeders. Vet. Microbiol..

[13] Beghain J., Bridier-Nahmias A., Le Nagard H., Denamur E., Clermont O. (2018). ClermonTyping: An Easy-to-Use and Accurate in silico Method for Escherichia Genus Strain Phylotyping. Microb. Genom..

[14] Mosquito S., Pons M.J., Riveros M., Ruiz J., Ochoa T.J. (2015). Diarrheagenic *Escherichia coli* Phylogroups Are Associated with Antibiotic Resistance and Duration of Diarrheal Episode. Sci. World J..

[15] Panth Y. (2019). Colibacillosis in Poultry: A Review. J. Agric. Nat. Resour..

[16] Kemmett K., Humphrey T., Rushton S., Close A., Wigley P., Williams N.J. (2013). A Longitudinal Study Simultaneously Exploring the Carriage of APEC Virulence Associated Genes and the Molecular Epidemiology of Faecal and Systemic *E. coli* in Commercial Broiler Chickens. PLoS ONE.

[17] Xiao S.-S., Mi J.-D., Mei L., Liang J., Feng K.-X., Wu Y.-B., Liao X.-D., Wang Y. (2021). Microbial Diversity and Community Variation in the Intestines of Layer Chickens. Animals.

[18] Yang M., Shi L., Ge Y., Leng D., Zeng B., Wang T., Jie H., Li D. (2022). Dynamic Changes in the Gut Microbial Community and Function during Broiler Growth. Microbiol. Spectr..

[19] Stromberg Z.R., Johnson J.R., Fairbrother J.M., Kilbourne J., Van Goor A., Curtiss R., Mellata M. (2017). Evaluation of *Escherichia coli* Isolates from Healthy Chickens to Determine Their Potential Risk to Poultry and Human Health. PLoS ONE.

[20] Zou M., Ma P.-P., Liu W.-S., Liang X., Li X.-Y., Li Y.-Z., Liu B.-T. (2021). Prevalence and Antibiotic Resistance Characteristics of Extraintestinal Pathogenic *Escherichia coli* among Healthy Chickens from Farms and Live Poultry Markets in China. Animals.

[21] Ewers C., Antão E.-M., Diehl I., Philipp H.-C., Wieler L.H. (2009). Intestine and Environment of the Chicken as Reservoirs for Extraintestinal Pathogenic *Escherichia coli* Strains with Zoonotic Potential. Appl. Environ. Microbiol..

[22] Dziva F., Stevens M.P. (2008). Colibacillosis in Poultry: Unravelling the Molecular Basis of Virulence of Avian Pathogenic *Escherichia coli* in Their Natural Hosts. Avian. Pathol..

[23] Malik Y.S., Milton A.A.P., Ghatak S., Ghosh S. (2021). Avian Colibacillosis (*Escherichia coli*). Role of Birds in Transmitting Zoonotic Pathogens.

[24] Wang X., Li L., Shang H., Zhou F., Wang C., Zhang S., Gao P., Guo P., Zhu R., Sun Z. (2022). Effects of Duck Circovirus on Immune Function and Secondary Infection of Avian Pathogenic *Escherichia coli*. Poult. Sci..

[25] Antão E.-M., Glodde S., Li G., Sharifi R., Homeier T., Laturnus C., Diehl I., Bethe A., Philipp H.-C., Preisinger R. (2008). The Chicken as a Natural Model for Extraintestinal Infections Caused by Avian Pathogenic *Escherichia coli* (APEC). Microb. Pathog..

[26] Kromann S., Olsen R.H., Bojesen A.M., Jensen H.E., Thøfner I. (2021). Development of an Aerogenous *Escherichia coli* Infection Model in Adult Broiler Breeders. Sci. Rep..

[27] Paudel S., Fink D., Abdelhamid M.K., Zöggeler A., Liebhart D., Hess M., Hess C. (2021). Aerosol Is the Optimal Route of Respiratory Tract Infection to Induce Pathological Lesions of Colibacillosis by a Lux-Tagged Avian Pathogenic *Escherichia coli* in Chickens. Avian Pathol..

[28] Christensen H., Bachmeier J., Bisgaard M. (2021). New Strategies to Prevent and Control Avian Pathogenic *Escherichia coli* (APEC). Avian Pathol..

[29] Joseph J., Zhang L., Adhikari P., Evans J.D., Ramachandran R. (2023). Avian Pathogenic *Escherichia coli* (APEC) in Broiler Breeders: An Overview. Pathogens.

[30] Mehat J.W., van Vliet A.H.M., La Ragione R.M. (2021). The Avian Pathogenic *Escherichia coli* (APEC) Pathotype Is Comprised of Multiple Distinct, Independent Genotypes. Avian Pathol..

[31] Kathayat D., Lokesh D., Ranjit S., Rajashekara G. (2021). Avian Pathogenic *Escherichia coli* (APEC): An Overview of Virulence and Pathogenesis Factors, Zoonotic Potential, and Control Strategies. Pathogens.

[32] Grakh K., Mittal D., Parkash A., Bangar Y.C. (2020). Assessing the Potential Risk Factors Associated with Avian Colibacillosis Using a Questionnaire Survey. Haryana Vet..

[33] Alber A., Stevens M.P., Vervelde L. (2021). The Bird’s Immune Response to Avian Pathogenic *Escherichia coli*. Avian Pathol..

[34] Arné P., Marc D., Brée A., Schouler C., Dho-Moulin M. (2000). Increased Tracheal Colonization in Chickens without Impairing Pathogenic Properties of Avian Pathogenic *Escherichia coli* MT78 with a *fimH* Deletion. Avian Dis..

[35] Ramírez R.M., Almanza Y., García S., Heredia N. (2009). Adherence and Invasion of Avian Pathogenic *Escherichia coli* to Avian Tracheal Epithelial Cells. World J. Microbiol. Biotechnol..

[36] Zhang G., Sunkara L.T. (2014). Avian Antimicrobial Host Defense Peptides: From Biology to Therapeutic Applications. Pharmaceuticals.

[37] Wu H., Xiong H., Huang X., Zhou Q., Hu D., Qi K., Liu H. (2022). Lung Infection of Avian Pathogenic *Escherichia coli* Co-Upregulates the Expression of CSP-A and CLL in Chickens. Res. Vet. Sci..

[38] Peng L.-Y., Cui Z.-Q., Wu Z.-M., Fu B.-D., Yi P.-F., Shen H.-Q. (2019). RNA-Seq Profiles of Chicken Type II Pneumocyte in Response to *Escherichia coli* Infection. PLoS ONE.

[39] Nawab A., An L., Wu J., Li G., Liu W., Zhao Y., Wu Q., Xiao M. (2019). Chicken Toll-like Receptors and Their Significance in Immune Response and Disease Resistance. Int. Rev. Immunol..

[40] Wigley P. (2013). Immunity to Bacterial Infection in the Chicken. Dev. Comp. Immunol..

[41] Reese S., Dalamani G., Kaspers B. (2006). The Avian Lung-Associated Immune System: A Review. Vet. Res..

[42] Sutton K., Costa T., Alber A., Bryson K., Borowska D., Balic A., Kaiser P., Stevens M., Vervelde L. (2018). Visualisation and Characterisation of Mononuclear Phagocytes in the Chicken Respiratory Tract Using CSF1R-Transgenic Chickens. Vet. Res..

[43] Maina J.N. (2023). A Critical Assessment of the Cellular Defences of the Avian Respiratory System: Are Birds in General and Poultry in Particular Relatively More Susceptible to Pulmonary Infections/Afflictions?. Biol. Rev..

[44] Ariaans M.P., Matthijs M.G.R., van Haarlem D., van de Haar P., van Eck J.H.H., Hensen E.J., Vervelde L. (2008). The Role of Phagocytic Cells in Enhanced Susceptibility of Broilers to Colibacillosis after Infectious Bronchitis Virus Infection. Vet. Immunol. Immunopathol..

[45] Li Z., Qi Z., Wang X., Lu L., Wang H., He Z., Chen Z., Shao Y., Tu J., Song X. (2023). Avian Pathogenic *Escherichia coli* Infection Causes Infiltration of Heterophilic Granulocytes of Chick Tracheal by the Complement and Coagulation Cascades Pathway. BMC Vet. Res..

[46] Alber A., Morris K.M., Bryson K.J., Sutton K.M., Monson M.S., Chintoan-Uta C., Borowska D., Lamont S.J., Schouler C., Kaiser P. (2019). Avian Pathogenic *Escherichia coli* (APEC) Strain-Dependent Immunomodulation of Respiratory Granulocytes and Mononuclear Phagocytes in CSF1R-Reporter Transgenic Chickens. Front. Immunol..

[47] Wigley P. (2021). The Immune System of the Chicken. Poultry Health: A Guide for Professionals.

[48] Van Goor A., Stromberg Z.R., Mellata M. (2017). A Recombinant Multi-Antigen Vaccine with Broad Protection Potential against Avian Pathogenic *Escherichia coli*. PLoS ONE.

[49] Ye X., Hsu C.-Y., Jia L., Zhang X., Magee C., Whitham S., Leigh S., Evans J.D., Zhang L., Robinson K. (2025). Dynamic Immune Response to Avian Pathogenic *Escherichia coli* Infection in Broiler Chickens: Insights into pro-Inflammatory and Anti-Inflammatory Cytokine Regulation. Poult. Sci..

[50] Bagheri S., Mitra T., Paudel S., Abdelhamid M.K., Könnyü S., Wijewardana V., Kangethe R.T., Cattoli G., Lyrakis M., Hess C. (2023). Aerosol Vaccination of Chicken Pullets with Irradiated Avian Pathogenic *Escherichia coli* Induces a Local Immunostimulatory Effect. Front. Immunol..

[51] Goonewardene K., Ahmed K.A., Gunawardana T., Popowich S., Kurukulasuriya S., Karunarathna R., Gupta A., Ayalew L.E., Lockerbie B., Foldvari M. (2020). Mucosal Delivery of CpG-ODN Mimicking Bacterial DNA via the Intrapulmonary Route Induces Systemic Antimicrobial Immune Responses in Neonatal Chicks. Sci. Rep..

[52] Mellata M., Dho-Moulin M., Dozois C.M., Curtiss R., Lehoux B., Fairbrother J.M. (2003). Role of Avian Pathogenic *Escherichia coli* Virulence Factors in Bacterial Interaction with Chicken Heterophils and Macrophages. Infect. Immun..

[53] Matter L.B., Barbieri N.L., Nordhoff M., Ewers C., Horn F. (2011). Avian Pathogenic *Escherichia coli* MT78 Invades Chicken Fibroblasts. Vet. Microbiol..

[54] Zhuge X., Sun Y., Jiang M., Wang J., Tang F., Xue F., Ren J., Zhu W., Dai J. (2019). Acetate Metabolic Requirement of Avian Pathogenic *Escherichia coli* Promotes Its Intracellular Proliferation within Macrophage. Vet. Res..

[55] Gao Q., Su S., Li X., Wang H., Liu J., Gao S. (2020). Transcriptional Analysis of RstA/RstB in Avian Pathogenic *Escherichia coli* Identifies Its Role in the Regulation of HdeD-Mediated Virulence and Survival in Chicken Macrophages. Vet. Microbiol..

[56] Horn F., Corrêa A.M.R., Barbieri N.L., Glodde S., Weyrauch K.D., Kaspers B., Driemeier D., Ewers C., Wieler L.H. (2012). Infections with Avian Pathogenic and Fecal *Escherichia coli* Strains Display Similar Lung Histopathology and Macrophage Apoptosis. PLoS ONE.

[57] Sharif H., Javed M.T., Ghafoor H., Younis M., Khan S.U., Rehman A.U., Ashfaq K., Saleem G., Manzoor F., Tariq N. (2018). Association of Pathogenicity Genes (*cvaC*, *iss*, *iutA*, *Stx1A*, *Stx2A* and *Vat*) of *E. coli* with Gross and Histopathological Lesions of Colibacillosis in Broilers. Sci. Lett..

[58] Shah S.A., Mir M.S., Wani B.M., Kamil S.A., Goswami P., Amin U., Shafi M., Rather M.A., Beigh A.B. (2019). Pathological Studies on Avian Pathogenic *Escherichia coli* Infection in Broilers. Pharma Innov. J..

[59] Joshua B.I., Nanchak L.R., Ayo A.C., Jummai B.D., Emmanuel L.L., Suleiman I., Alesa U.M., Dominic U.A., Pwajok C.T.P., Gunya D.Y. (2022). Gross and Histopatholigical Lesions Associated with *Escherichia coli* Infection in Chickens Examined, At Ecwa Veterinary Clinic, Bukuru, Plateau State, Nigeria. Acta Sci. Microbiol..

[60] Nolan L.K., Barnes H.J., Vaillancourt J.-P., Abdul-Aziz T., Logue C.M., Swayne D.E. (2013). Colibacillosis. Diseases of Poultry.

[61] Lutful Kabir S.M., Sikder M.H., Alam J., Neogi S.B., Yamasaki S., Hester P.Y. (2017). Colibacillosis and Its Impact on Egg Production. Egg Innovations and Strategies for Improvements.

[62] Gerasimova A.O., Novikova O.B., Savicheva A.A. (2023). Avian Colibacillosis—Current Aspects. Vet. Sci. Today.

[63] Wang C., Pors S.E., Olsen R.H., Bojesen A.M. (2018). Transmission and Pathogenicity of *Gallibacterium anatis* and *Escherichia coli* in Embryonated Eggs. Vet. Microbiol..

[64] Oikarainen P.E., Pohjola L.K., Pietola E.S., Heikinheimo A. (2019). Direct Vertical Transmission of ESBL/PAmpC-Producing *Escherichia coli* Limited in Poultry Production Pyramid. Vet. Microbiol..

[65] Swelum A.A., Elbestawy A.R., El-Saadony M.T., Hussein E.O.S., Alhotan R., Suliman G.M., Taha A.E., Ba-Awadh H., El-Tarabily K.A., Abd El-Hack M.E. (2021). Ways to Minimize Bacterial Infections, with Special Reference to *Escherichia coli*, to Cope with the First-Week Mortality in Chicks: An Updated Overview. Poult. Sci..

[66] Govindarajan D.K., Viswalingam N., Meganathan Y., Kandaswamy K. (2020). Adherence Patterns of *Escherichia coli* in the Intestine and Its Role in Pathogenesis. Med. Microecol..

[67] Solanki V., Tiwari M., Tiwari V. (2018). Host-Bacteria Interaction and Adhesin Study for Development of Therapeutics. Int. J. Biol. Macromol..

[68] Aleksandrowicz A., Khan M.M., Sidorczuk K., Noszka M., Kolenda R. (2021). Whatever Makes Them Stick—Adhesins of Avian Pathogenic *Escherichia coli*. Vet. Microbiol..

[69] Isidro-Coxca M.I., Ortiz-Jiménez S., Puente J.L. (2024). Type 1 Fimbria and P Pili: Regulatory Mechanisms of the Prototypical Members of the Chaperone-Usher Fimbrial Family. Arch. Microbiol..

[70] LA Ragione R.M., Cooley W.A., Woodward M.J. (2000). The Role of Fimbriae and Flagella in the Adherence of Avian Strains of *Escherichia coli* O78:K80 to Tissue Culture Cells and Tracheal and Gut Explants. J. Med. Microbiol..

[71] Chen J., Dai W., Cui S., Lei W., Dai D. (2023). Screening of Antigenic Epitopes Related to the Adhesion of the Avian *Escherichia coli* Type 1 Fimbrial Agglutinin Domain. BMC Vet. Res..

[72] Kline K.A., Fälker S., Dahlberg S., Normark S., Henriques-Normark B. (2009). Bacterial Adhesins in Host-Microbe Interactions. Cell Host Microbe.

[73] Avalos Vizcarra I., Hosseini V., Kollmannsberger P., Meier S., Weber S.S., Arnoldini M., Ackermann M., Vogel V. (2016). How Type 1 Fimbriae Help *Escherichia coli* to Evade Extracellular Antibiotics. Sci. Rep..

[74] Nawaz S., Wang Z., Zhang Y., Jia Y., Jiang W., Chen Z., Yin H., Huang C., Han X. (2024). Avian Pathogenic *Escherichia coli* (APEC): Current Insights and Future Challenges. Poult. Sci..

[75] Werneburg G.T., Thanassi D.G. (2018). Pili Assembled by the Chaperone/Usher Pathway in *Escherichia coli* and *Salmonella*. EcoSal Plus.

[76] Lane M.C., Mobley H.L.T. (2007). Role of P-Fimbrial-Mediated Adherence in Pyelonephritis and Persistence of Uropathogenic *Escherichia coli* (UPEC) in the Mammalian Kidney. Kidney Int..

[77] Ghazvini H., Taheri K., Edalati E., Sedighi M., Mirkalantari S. (2019). Virulence Factors and Antimicrobial Resistance in Uropathogenic *Escherichia coli* Strains Isolated from Cystitis and Pyelonephritis. Turk. J. Med. Sci..

[78] Rocha A.C.G.P., Rocha S.L.S., Lima-Rosa C.A.V., Souza G.F., Moraes H.L.S., Salle F.O., Moraes L.B., Salle C.T.P. (2008). Genes Associated with Pathogenicity of Avian *Escherichia coli* (APEC) Isolated from Respiratory Cases of Poultry. Pesq. Vet. Bras..

[79] Paixão A.C., Ferreira A.C., Fontes M., Themudo P., Albuquerque T., Soares M.C., Fevereiro M., Martins L., de Sá M.I.C. (2016). Detection of Virulence-Associated Genes in Pathogenic and Commensal Avian *Escherichia coli* Isolates. Poult. Sci..

[80] Xu X., Sun Q., Zhao L. (2019). Virulence Factors and Antibiotic Resistance of Avian Pathogenic *Escherichia coli* in Eastern China. J. Vet. Res..

[81] Kariyawasam S., Nolan L.K. (2009). Pap Mutant of Avian Pathogenic *Escherichia coli* O1, an O1:K1:H7 Strain, Is Attenuated in vivo. Avian Dis..

[82] Ganz T. (2018). Iron and Infection. Int. J. Hematol..

[83] Kramer J., Özkaya Ö., Kümmerli R. (2020). Bacterial Siderophores in Community and Host Interactions. Nat. Rev. Microbiol..

[84] Schalk I.J. (2025). Bacterial Siderophores: Diversity, Uptake Pathways and Applications. Nat. Rev. Microbiol..

[85] Page M.G.P. (2019). The Role of Iron and Siderophores in Infection, and the Development of Siderophore Antibiotics. Clin. Infect. Dis..

[86] Gatsios A., Kim C.S., Crawford J.M. (2021). *Escherichia coli* Small Molecule Metabolism at the Host–Microorganism Interface. Nat. Chem. Biol..

[87] Khasheii B., Mahmoodi P., Mohammadzadeh A. (2021). Siderophores: Importance in Bacterial Pathogenesis and Applications in Medicine and Industry. Microbiol. Res..

[88] Mey A.R., Gómez-Garzón C., Payne S.M. (2021). Iron Transport and Metabolism in *Escherichia, Shigella*, and *Salmonella*. EcoSal Plus.

[89] Moran R.A., Holt K.E., Hall R.M. (2016). pCERC3 from a Commensal ST95 *Escherichia coli*: A ColV Virulence-Multiresistance Plasmid Carrying a *Sul3*-Associated Class 1 Integron. Plasmid.

[90] Gao H., Bian X. (2022). Editorial: Microbial Siderophores: Biosynthesis, Regulation, and Physiological and Ecological Impacts. Front. Microbiol..

[91] Cui J., Dong Y., Chen Q., Zhang C., He K., Hu G., He D., Yuan L. (2024). Horizontal Transfer Characterization of ColV Plasmids in BlaCTX-M-Bearing Avian *Escherichia coli*. Poult. Sci..

[92] Ling J., Pan H., Gao Q., Xiong L., Zhou Y., Zhang D., Gao S., Liu X. (2013). Aerobactin Synthesis Genes *IucA* and *IucC* Contribute to the Pathogenicity of Avian Pathogenic *Escherichia coli* O2 Strain E058. PLoS ONE.

[93] Gao Q., Jia X., Wang X., Xiong L., Gao S., Liu X. (2015). The Avian Pathogenic *Escherichia coli* O2 Strain E058 Carrying the Defined Aerobactin-Defective *IucD* or *IucDiutA* Mutation Is Less Virulent in the Chicken. Infect. Genet. Evol..

[94] Perry R.D., Fetherston J.D. (2011). Yersiniabactin Iron Uptake: Mechanisms and Role in *Yersinia Pestis* Pathogenesis. Microbes Infect..

[95] Desvaux M., Dalmasso G., Beyrouthy R., Barnich N., Delmas J., Bonnet R. (2020). Pathogenicity Factors of Genomic Islands in Intestinal and Extraintestinal *Escherichia coli*. Front. Microbiol..

[96] Katumba G.L., Tran H., Henderson J.P. (2022). The *Yersinia* High-Pathogenicity Island Encodes a Siderophore-Dependent Copper Response System in Uropathogenic *Escherichia coli*. mBio.

[97] Miller D.A., Luo L., Hillson N., Keating T.A., Walsh C.T. (2002). Yersiniabactin Synthetase: A Four-Protein Assembly Line Producing the Nonribosomal Peptide/Polyketide Hybrid Siderophore of *Yersinia Pestis*. Chem. Biol..

[98] Schubert S., Rakin A., Heesemann J. (2004). The *Yersinia* High-Pathogenicity Island (HPI): Evolutionary and Functional Aspects. Int. J. Med. Microbiol..

[99] Schubert S., Picard B., Gouriou S., Heesemann J., Denamur E. (2002). *Yersinia* High-Pathogenicity Island Contributes to Virulence in *Escherichia coli* Causing Extraintestinal Infections. Infect. Immun..

[100] Johnson J.R., Magistro G., Clabots C., Porter S., Manges A., Thuras P., Schubert S. (2018). Contribution of Yersiniabactin to the Virulence of an *Escherichia coli* Sequence Type 69 (“Clonal Group A”) Cystitis Isolate in Murine Models of Urinary Tract Infection and Sepsis. Microb. Pathog..

[101] Brumbaugh A.R., Smith S.N., Subashchandrabose S., Himpsl S.D., Hazen T.H., Rasko D.A., Mobley H.L.T. (2015). Blocking Yersiniabactin Import Attenuates Extraintestinal Pathogenic *Escherichia coli* in Cystitis and Pyelonephritis and Represents a Novel Target to Prevent Urinary Tract Infection. Infect. Immun..

[102] Tu J., Xue T., Qi K., Shao Y., Huang B., Wang X., Zhou X. (2016). The Irp2 and FyuA Genes in High Pathogenicity Islands Are Involved in the Pathogenesis of Infections Caused by Avian Pathogenic *Escherichia coli* (APEC). Pol. J. Vet. Sci..

[103] Sorsa L.J., Dufke S., Heesemann J., Schubert S. (2003). Characterization of an *IroBCDEN* Gene Cluster on a Transmissible Plasmid of Uropathogenic *Escherichia coli*: Evidence for Horizontal Transfer of a Chromosomal Virulence Factor. Infect. Immun..

[104] Soliman A.M., Ramadan H., Yu L., Hisatsune J., Sugai M., Elnahriry S.S., Nariya H., El-Domany R.A., Shimamoto T., Jackson C.R. (2022). Complete Genome Sequences of Two *Escherichia coli* Clinical Isolates from Egypt Carrying *mcr-1* on IncP and IncX4 Plasmids. Front. Microbiol..

[105] Hantke K., Nicholson G., Rabsch W., Winkelmann G. (2003). Salmochelins, Siderophores of *Salmonella enterica* and Uropathogenic *Escherichia coli* Strains, Are Recognized by the Outer Membrane Receptor IroN. Proc. Natl. Acad. Sci. USA.

[106] Ons E., Bleyen N., Tuntufye H.N., Vandemaele F., Goddeeris B.M. (2007). High Prevalence Iron Receptor Genes of Avian Pathogenic *Escherichia coli*. Avian Pathol..

[107] Hossain F.E., Islam S., Islam M.A., Islam S., Ahmed F. (2021). Detection of Virulence Genes of APEC (Avian Pathogenic *Escherichia coli*) Isolated from Poultry in Noakhali, Bangladesh. Bioresearch Commun..

[108] Caza M., Lépine F., Milot S., Dozois C.M. (2008). Specific Roles of the IroBCDEN Genes in Virulence of an Avian Pathogenic *Escherichia coli* O78 Strain and in Production of Salmochelins. Infect. Immun..

[109] Gao Q., Wang X., Xu H., Xu Y., Ling J., Zhang D., Gao S., Liu X. (2012). Roles of Iron Acquisition Systems in Virulence of Extraintestinal Pathogenic *Escherichia coli*: Salmochelin and Aerobactin Contribute More to Virulence than Heme in a Chicken Infection Model. BMC Microbiol..

[110] Magistro G., Hoffmann C., Schubert S. (2015). The Salmochelin Receptor IroN Itself, but Not Salmochelin-Mediated Iron Uptake Promotes Biofilm Formation in Extraintestinal Pathogenic *Escherichia coli* (ExPEC). Int. J. Med. Microbiol..

[111] Silveira F., Maluta R.P., Tiba M.R., de Paiva J.B., Guastalli E.A.L., da Silveira W.D. (2016). Comparison between Avian Pathogenic (APEC) and Avian Faecal (AFEC) *Escherichia coli* Isolated from Different Regions in Brazil. Vet. J..

[112] Huja S., Oren Y., Trost E., Brzuszkiewicz E., Biran D., Blom J., Goesmann A., Gottschalk G., Hacker J., Ron E.Z. (2015). Genomic Avenue to Avian Colisepticemia. mBio.

[113] Xu W.-Y., Li Y.-J., Fan C. (2018). Different Loci and MRNA Copy Number of the Increased Serum Survival Gene of *Escherichia coli*. Can. J. Microbiol..

[114] Biran D., Sura T., Otto A., Yair Y., Becher D., Ron E.Z. (2021). Surviving Serum: The *Escherichia coli iss* Gene of Extraintestinal Pathogenic *E. coli* Is Required for the Synthesis of Group 4 Capsule. Infect. Immun..

[115] Nolan L.K., Horne S.M., Giddings C.W., Foley S.L., Johnson T.J., Lynne A.M., Skyberg J. (2003). Resistance to Serum Complement, *iss*, and Virulence of Avian *Escherichia coli*. Vet. Res. Commun..

[116] Lynne A.M., Skyberg J.A., Logue C.M., Nolan L.K. (2007). Detection of *iss* and *bor* on the Surface of *Escherichia coli*. J. Appl. Microbiol..

[117] Varga C., Brash M.L., Slavic D., Boerlin P., Ouckama R., Weis A., Petrik M., Philippe C., Barham M., Guerin M.T. (2018). Evaluating Virulence-Associated Genes and Antimicrobial Resistance of Avian Pathogenic *Escherichia coli* Isolates from Broiler and Broiler Breeder Chickens in Ontario, Canada. Avian Dis..

[118] Afayibo D.J.A., Zhu H., Zhang B., Yao L., Abdelgawad H.A., Tian M., Qi J., Liu Y., Wang S. (2022). Isolation, Molecular Characterization, and Antibiotic Resistance of Avian Pathogenic *Escherichia coli* in Eastern China. Vet. Sci..

[119] Wilczyński J., Stępień-Pyśniak D., Wystalska D., Wernicki A. (2022). Molecular and Serological Characteristics of Avian Pathogenic *Escherichia coli* Isolated from Various Clinical Cases of Poultry Colibacillosis in Poland. Animals.

[120] Wijetunge D.S.S., Gongati S., DebRoy C., Kim K.S., Couraud P.O., Romero I.A., Weksler B., Kariyawasam S. (2015). Characterizing the Pathotype of Neonatal Meningitis Causing *Escherichia coli* (NMEC). BMC Microbiol..

[121] Zeng Q., Xiao S., Gu F., He W., Xie Q., Yu F., Han L. (2021). Antimicrobial Resistance and Molecular Epidemiology of Uropathogenic *Escherichia coli* Isolated From Female Patients in Shanghai, China. Front. Cell. Infect. Microbiol..

[122] Chuba P.J., Leon M.A., Banerjee A., Palchaudhuri S. (1989). Cloning and DNA Sequence of Plasmid Determinant *iss*, Coding for Increased Serum Survival and Surface Exclusion, Which Has Homology with Lambda DNA. Mol. Gen. Genet..

[123] Miajlovic H., Smith S.G. (2014). Bacterial Self-Defence: How *Escherichia coli* Evades Serum Killing. FEMS Microbiol. Lett..

[124] Johnson T.J., Wannemuehler Y., Doetkott C., Johnson S.J., Rosenberger S.C., Nolan L.K. (2008). Identification of Minimal Predictors of Avian Pathogenic *Escherichia coli* Virulence for Use as a Rapid Diagnostic Tool. J. Clin. Microbiol..

[125] Pokharel P., Dhakal S., Dozois C.M. (2023). The Diversity of *Escherichia coli* Pathotypes and Vaccination Strategies against This Versatile Bacterial Pathogen. Microorganisms.

[126] Kariyawasam S., Han J. (2019). Avian Pathogenic *Escherichia coli*: Link to Foodborne Urinary Tract Infections in Humans. Food Safety in Poultry Meat Production.

[127] Rodriguez-Siek K.E., Giddings C.W., Doetkott C., Johnson T.J., Fakhr M.K., Nolan L.K. (2005). Comparison of *Escherichia coli* Isolates Implicated in Human Urinary Tract Infection and Avian Colibacillosis. Microbiology.

[128] Ron E.Z. (2006). Host Specificity of Septicemic *Escherichia coli*: Human and Avian Pathogens. Curr. Opin. Microbiol..

[129] Adiri R.S., Gophna U., Ron E.Z. (2003). Multilocus Sequence Typing (MLST) of *Escherichia coli* O78 Strains. FEMS Microbiol. Lett..

[130] Johnson T.J., Wannemuehler Y., Johnson S.J., Stell A.L., Doetkott C., Johnson J.R., Kim K.S., Spanjaard L., Nolan L.K. (2008). Comparison of Extraintestinal Pathogenic *Escherichia coli* Strains from Human and Avian Sources Reveals a Mixed Subset Representing Potential Zoonotic Pathogens. Appl. Environ. Microbiol..

[131] Mora A., Viso S., López C., Alonso M.P., García-Garrote F., Dabhi G., Mamani R., Herrera A., Marzoa J., Blanco M. (2013). Poultry as Reservoir for Extraintestinal Pathogenic *Escherichia coli* O45:K1:H7-B2-ST95 in Humans. Vet. Microbiol..

[132] Danzeisen J.L., Wannemuehler Y., Nolan L.K., Johnson T.J. (2013). Comparison of Multilocus Sequence Analysis and Virulence Genotyping of *Escherichia coli* from Live Birds, Retail Poultry Meat, and Human Extraintestinal Infection. Avian Dis..

[133] Maluta R.P., Logue C.M., Casas M.R.T., Meng T., Guastalli E.A.L., Rojas T.C.G., Montelli A.C., Sadatsune T., de Carvalho Ramos M., Nolan L.K. (2014). Overlapped Sequence Types (STs) and Serogroups of Avian Pathogenic (APEC) and Human Extra-Intestinal Pathogenic (ExPEC) *Escherichia coli* Isolated in Brazil. PLoS ONE.

[134] Hojabri Z., Darabi N., Arab M., Saffari F., Pajand O. (2019). Clonal Diversity, Virulence Genes Content and Subclone Status of *Escherichia coli* Sequence Type 131: Comparative Analysis of *E. coli* ST131 and Non-ST131 Isolates from Iran. BMC Microbiol..

[135] Massella E., Reid C.J., Cummins M.L., Anantanawat K., Zingali T., Serraino A., Piva S., Giacometti F., Djordjevic S.P. (2020). Snapshot Study of Whole Genome Sequences of *Escherichia coli* from Healthy Companion Animals, Livestock, Wildlife, Humans and Food in Italy. Antibiotics.

[136] Díaz-Jiménez D., García-Meniño I., Fernández J., García V., Mora A. (2020). Chicken and Turkey Meat: Consumer Exposure to Multidrug-Resistant Enterobacteriaceae Including Mcr-Carriers, Uropathogenic *E. coli* and High-Risk Lineages Such as ST131. Int. J. Food Microbiol..

[137] Moulin-Schouleur M., Schouler C., Tailliez P., Kao M.-R., Brée A., Germon P., Oswald E., Mainil J., Blanco M., Blanco J. (2006). Common Virulence Factors and Genetic Relationships between O18:K1:H7 *Escherichia coli* Isolates of Human and Avian Origin. J. Clin. Microbiol..

[138] Moulin-Schouleur M., Répérant M., Laurent S., Brée A., Mignon-Grasteau S., Germon P., Rasschaert D., Schouler C. (2007). Extraintestinal Pathogenic *Escherichia coli* Strains of Avian and Human Origin: Link between Phylogenetic Relationships and Common Virulence Patterns. J. Clin. Microbiol..

[139] Jakobsen L., Hammerum A.M., Frimodt-Møller N. (2010). Virulence of *Escherichia coli* B2 Isolates from Meat and Animals in a Murine Model of Ascending Urinary Tract Infection (UTI): Evidence That UTI Is a Zoonosis. J. Clin. Microbiol..

[140] Tivendale K.A., Logue C.M., Kariyawasam S., Jordan D., Hussein A., Li G., Wannemuehler Y., Nolan L.K. (2010). Avian-Pathogenic *Escherichia coli* Strains Are Similar to Neonatal Meningitis *E. coli* Strains and Are Able to Cause Meningitis in the Rat Model of Human Disease. Infect. Immun..

[141] Chanteloup N.K., Porcheron G., Delaleu B., Germon P., Schouler C., Moulin-Schouleur M., Gilot P. (2011). The Extra-Intestinal Avian Pathogenic *Escherichia coli* Strain BEN2908 Invades Avian and Human Epithelial Cells and Survives Intracellularly. Vet. Microbiol..

[142] Wang X., Wei L., Wang B., Zhang R., Liu C., Bi D., Chen H., Tan C. (2016). Complete Genome Sequence and Characterization of Avian Pathogenic *Escherichia coli* Field Isolate ACN001. Stand. Genomic. Sci..

[143] Mellata M., Johnson J.R., Curtiss R. (2018). *Escherichia coli* Isolates from Commercial Chicken Meat and Eggs Cause Sepsis, Meningitis and Urinary Tract Infection in Rodent Models of Human Infections. Zoonoses Public Health.

[144] Johnson T.J., Kariyawasam S., Wannemuehler Y., Mangiamele P., Johnson S.J., Doetkott C., Skyberg J.A., Lynne A.M., Johnson J.R., Nolan L.K. (2007). The Genome Sequence of Avian Pathogenic *Escherichia coli* Strain O1:K1:H7 Shares Strong Similarities with Human Extraintestinal Pathogenic *E. coli* Genomes. J. Bacteriol..

[145] Pietsch M., Irrgang A., Roschanski N., Brenner Michael G., Hamprecht A., Rieber H., Käsbohrer A., Schwarz S., Rösler U., Kreienbrock L. (2018). Whole Genome Analyses of CMY-2-Producing *Escherichia coli* Isolates from Humans, Animals and Food in Germany. BMC Genom..

[146] Li D., Wyrsch E.R., Elankumaran P., Dolejska M., Marenda M.S., Browning G.F., Bushell R.N., McKinnon J., Chowdhury P.R., Hitchick N. (2021). Genomic Comparisons of *Escherichia coli* ST131 from Australia. Microb. Genom..

[147] Tsai Y.-Y., Ienes Lima J., Alvarez Narvaez S., Logue C.M. (2025). Whole-Genome Analysis of Five *Escherichia coli* Strains Isolated from Focal Duodenal Necrosis in Laying Hens Reveals Genetic Similarities to the *E. coli* O25:H4 ST131 Strain. Microbiol. Spectr..

[148] Xia F., Cheng J., Jiang M., Wang Z., Wen Z., Wang M., Ren J., Zhuge X. (2022). Genomics Analysis to Identify Multiple Genetic Determinants That Drive the Global Transmission of the Pandemic ST95 Lineage of Extraintestinal Pathogenic *Escherichia coli* (ExPEC). Pathogens.

[149] Saidenberg A.B.S., Edslev S.M., Hallstrøm S., Rasmussen A., Park D.E., Aziz M., Dos Santos Queiroz B., Baptista A.A.S., Barbosa F., Rocha V.G.P. (2024). *Escherichia coli* ST117: Exploring the Zoonotic Hypothesis. Microbiol. Spectr..

[150] Guabiraba R., Schouler C. (2015). Avian Colibacillosis: Still Many Black Holes. FEMS Microbiol. Lett..

[151] Kunert Filho H.C., Carvalho D., Grassotti T.T., Soares B.D., Rossato J.M., Cunha A.C., Brito K.C.T., Cavalli L.S., Brito B.G. (2015). Avian Pathogenic *Escherichia coli*—Methods for Improved Diagnosis. Worlds Poult. Sci. J..

[152] Younis G., Awad A., Mohamed N. (2017). Phenotypic and Genotypic Characterization of Antimicrobial Susceptibility of Avian Pathogenic *Escherichia coli* Isolated from Broiler Chickens. Vet. World.

[153] Kim Y.B., Yoon M.Y., Ha J.S., Seo K.W., Noh E.B., Son S.H., Lee Y.J. (2020). Molecular Characterization of Avian Pathogenic *Escherichia coli* from Broiler Chickens with Colibacillosis. Poult. Sci..

[154] Hu J., Afayibo D.J.A., Zhang B., Zhu H., Yao L., Guo W., Wang X., Wang Z., Wang D., Peng H. (2022). Characteristics, Pathogenic Mechanism, Zoonotic Potential, Drug Resistance, and Prevention of Avian Pathogenic *Escherichia coli* (APEC). Front. Microbiol..

[155] Hussain H.I., Iqbal Z., Iqbal M., Kuang X., Wang Y., Yang L., Ihsan A., Aqib A.I., Kaleem Q.M., Gu Y. (2022). Coexistence of Virulence and β-Lactamase Genes in Avian Pathogenic *Escherichia coli*. Microb. Pathog..

[156] Collingwood C., Kemmett K., Williams N., Wigley P. (2014). Is the Concept of Avian Pathogenic *Escherichia coli* as a Single Pathotype Fundamentally Flawed?. Front. Vet. Sci..

[157] Feng A., Akter S., Leigh S.A., Wang H., Pharr G.T., Evans J., Branton S.L., Landinez M.P., Pace L., Wan X.-F. (2023). Genomic Diversity, Pathogenicity and Antimicrobial Resistance of *Escherichia coli* Isolated from Poultry in the Southern United States. BMC Microbiol..

[158] La Ragione R.M., Woodward M.J. (2002). Virulence Factors of *Escherichia coli* Serotypes Associated with Avian Colisepticaemia. Res. Vet. Sci..

[159] Delicato E.R., de Brito B.G., Gaziri L.C.J., Vidotto M.C. (2003). Virulence-Associated Genes in *Escherichia coli* Isolates from Poultry with Colibacillosis. Vet. Microbiol..

[160] Mohamed L., Ge Z., Yuehua L., Yubin G., Rachid K., Mustapha O., Junwei W., Karine O. (2018). Virulence Traits of Avian Pathogenic (APEC) and Fecal (AFEC) *E. coli* Isolated from Broiler Chickens in Algeria. Trop. Anim. Health Prod..

[161] Subedi M., Luitel H., Devkota B., Bhattarai R.K., Phuyal S., Panthi P., Shrestha A., Chaudhary D.K. (2018). Antibiotic Resistance Pattern and Virulence Genes Content in Avian Pathogenic *Escherichia coli* (APEC) from Broiler Chickens in Chitwan, Nepal. BMC Vet. Res..

[162] Johar A., Al-Thani N., Al-Hadidi S.H., Dlissi E., Mahmoud M.H., Eltai N.O. (2021). Antibiotic Resistance and Virulence Gene Patterns Associated with Avian Pathogenic *Escherichia coli* (APEC) from Broiler Chickens in Qatar. Antibiotics.

[163] Skyberg J.A., Horne S.M., Giddings C.W., Wooley R.E., Gibbs P.S., Nolan L.K. (2003). Characterizing Avian *Escherichia coli* Isolates with Multiplex Polymerase Chain Reaction. Avian Dis..

[164] Ewers C., Janssen T., Kiessling S., Philipp H.-C., Wieler L.H. (2005). Rapid Detection of Virulence-Associated Genes in Avian Pathogenic *Escherichia coli* by Multiplex Polymerase Chain Reaction. Avian Dis..

[165] De Carli S., Ikuta N., Lehmann F.K.M., da Silveira V.P., de Melo Predebon G., Fonseca A.S.K., Lunge V.R. (2015). Virulence Gene Content in *Escherichia coli* Isolates from Poultry Flocks with Clinical Signs of Colibacillosis in Brazil. Poult. Sci..

[166] Ovi F., Zhang L., Nabors H., Jia L., Adhikari P. (2023). A Compilation of Virulence-Associated Genes That Are Frequently Reported in Avian Pathogenic *Escherichia coli* (APEC) Compared to Other *E. coli*. J. Appl. Microbiol..

[167] Messaili C., Messai Y., Bakour R. (2019). Virulence Gene Profiles, Antimicrobial Resistance and Phylogenetic Groups of Fecal *Escherichia coli* Strains Isolated from Broiler Chickens in Algeria. Vet. Ital..

[168] Kazimierczak J., Pospiech K., Sowińska P., Pękala A., Borówka P., Wójcik E.A., Marciniak B., Lis M.W., Strapagiel D., Dastych J. (2025). A Rapid Detection of Avian Pathogenic *Escherichia coli* (APEC) Strains Based on Minimal Number of Virulence Markers Identified by Whole Genome Sequencing. BMC Microbiol..

[169] Johnson T.J., Siek K.E., Johnson S.J., Nolan L.K. (2006). DNA Sequence of a ColV Plasmid and Prevalence of Selected Plasmid-Encoded Virulence Genes among Avian *Escherichia coli* Strains. J. Bacteriol..

[170] Papoušková A., Čížek A. (2020). A Complex Approach to a Complex Problem: The Use of Whole-Genome Sequencing in Monitoring Avian-Pathogenic *Escherichia coli*—A Review. Acta Vet. Brno.

[171] Johnson T.J., Miller E.A., Flores-Figueroa C., Munoz-Aguayo J., Cardona C., Fransen K., Lighty M., Gonder E., Nezworski J., Haag A. (2022). Refining the Definition of the Avian Pathogenic *Escherichia coli* (APEC) Pathotype through Inclusion of High-Risk Clonal Groups. Poult. Sci..

[172] Cummins M.L., Reid C.J., Roy Chowdhury P., Bushell R.N., Esbert N., Tivendale K.A., Noormohammadi A.H., Islam S., Marenda M.S., Browning G.F. (2019). Whole Genome Sequence Analysis of Australian Avian Pathogenic *Escherichia coli* That Carry the Class 1 Integrase Gene. Microb. Genom..

[173] Cordoni G., Woodward M.J., Wu H., Alanazi M., Wallis T., La Ragione R.M. (2016). Comparative Genomics of European Avian Pathogenic *E. coli* (APEC). BMC Genom..

[174] Azam M., Mohsin M., Johnson T.J., Smith E.A., Johnson A., Umair M., Saleemi M.K. (2020). Genomic Landscape of Multi-Drug Resistant Avian Pathogenic *Escherichia coli* Recovered from Broilers. Vet. Microbiol..

[175] Chen X., Liu W., Li H., Yan S., Jiang F., Cai W., Li G. (2021). Whole Genome Sequencing Analysis of Avian Pathogenic *Escherichia coli* from China. Vet. Microbiol..

[176] Papouskova A., Masarikova M., Valcek A., Senk D., Cejkova D., Jahodarova E., Cizek A. (2020). Genomic Analysis of *Escherichia coli* Strains Isolated from Diseased Chicken in the Czech Republic. BMC Vet. Res..

[177] Poulsen L.L., Kudirkiene E., Jørgensen S.L., Djordjevic S.P., Cummins M.L., Christensen J.P., Christensen H., Bisgaard M., Thøfner I. (2020). Whole Genome Sequence Comparison of Avian Pathogenic *Escherichia coli* from Acute and Chronic Salpingitis of Egg Laying Hens. BMC Vet. Res..

[178] Rafique M., Potter R.F., Ferreiro A., Wallace M.A., Rahim A., Ali Malik A., Siddique N., Abbas M.A., D’Souza A.W., Burnham C.-A.D. (2019). Genomic Characterization of Antibiotic Resistant *Escherichia coli* Isolated from Domestic Chickens in Pakistan. Front. Microbiol..

[179] Mageiros L., Méric G., Bayliss S.C., Pensar J., Pascoe B., Mourkas E., Calland J.K., Yahara K., Murray S., Wilkinson T.S. (2021). Genome Evolution and the Emergence of Pathogenicity in Avian *Escherichia coli*. Nat. Commun..

[180] Weerts E.A.W.S., Matthijs M.G.R., Bonhof J., van Haarlem D.A., Dwars R.M., Gröne A., Verheije M.H., Jansen C.A. (2021). The Contribution of the Immune Response to Enhanced Colibacillosis upon Preceding Viral Respiratory Infection in Broiler Chicken in a Dual Infection Model. Vet. Immunol. Immunopathol..

[181] Liu Q.X., Zhou Y., Li X.M., Ma D.D., Xing S., Feng J.H., Zhang M.H. (2020). Ammonia Induce Lung Tissue Injury in Broilers by Activating NLRP3 Inflammasome via *Escherichia/Shigella*. Poult. Sci..

[182] Sheikh I.U., Nissa S.S., Zaffer B., Bulbul K.H., Akand A.H., Ahmed H.A., Hasin D., Hussain I., Hussain S.A. (2018). Ammonia Production in the Poultry Houses and Its Harmful Effects. Int. J. Vet. Sci. Anim. Husb..

[183] Prabakar G., Pavulraj S., Shanmuganathan S., Kirubakaran A., Mohana N. (2016). Early Nutrition and Its Importance in Poultry: A Review. Indian J. Anim. Nutr..

[184] Ghimpețeanu O.M., Pogurschi E.N., Popa D.C., Dragomir N., Drăgotoiu T., Mihai O.D., Petcu C.D. (2022). Antibiotic Use in Livestock and Residues in Food-A Public Health Threat: A Review. Foods.

[185] Vermeulen B., De Backer P., Remon J.P. (2002). Drug Administration to Poultry. Adv. Drug Deliv. Rev..

[186] Agunos A., Léger D., Carson C. (2012). Review of Antimicrobial Therapy of Selected Bacterial Diseases in Broiler Chickens in Canada. Can. Vet. J..

[187] Glisson J.R., Hofacre C.L., Mathis G.F. (2004). Comparative Efficacy of Enrofloxacin, Oxytetracycline, and Sulfadimethoxine for the Control of Morbidity and Mortality Caused by *Escherichia coli* in Broiler Chickens. Avian Dis..

[188] Akbar H., Khan M., Khan A.A., Khan M.A., Shuaib M., Akbar S.F., Manzoor S., Irshad-ur-rehman, Ahmad S., Ali L. (2009). Comparative Efficacy of Doxycycline and Flumequine Against Experimentally Induced Colibacillosis in Broiler Chicks. J. Vet. Med. Anim. Health.

[189] Dheilly A., Bouder A., Le Devendec L., Hellard G., Kempf I. (2011). Clinical and Microbial Efficacy of Antimicrobial Treatments of Experimental Avian Colibacillosis. Vet. Microbiol..

[190] Haq K.U., Khan A.A., Ullah S., Nabi G. (2015). Comparative Efficacy of Norfloxacin, Clarithromycin and Cefpodoxime Against Experimentally Induced Colibacillosis in Pigeons. Am. Eurasian J. Toxicol. Sci..

[191] Escolà-Vergé L., Los-Arcos I., Almirante B. (2020). New Antibiotics for the Treatment of Infections by Multidrug-Resistant Microorganisms. Med. Clin..

[192] Brown K., Uwiera R.R.E., Kalmokoff M.L., Brooks S.P.J., Inglis G.D. (2017). Antimicrobial Growth Promoter Use in Livestock: A Requirement to Understand Their Modes of Action to Develop Effective Alternatives. Int. J. Antimicrob. Agents.

[193] Kalia V.C., Shim W.Y., Patel S.K.S., Gong C., Lee J.-K. (2022). Recent Developments in Antimicrobial Growth Promoters in Chicken Health: Opportunities and Challenges. Sci. Total Environ..

[194] Abreu R., Semedo-Lemsaddek T., Cunha E., Tavares L., Oliveira M. (2023). Antimicrobial Drug Resistance in Poultry Production: Current Status and Innovative Strategies for Bacterial Control. Microorganisms.

[195] Agyare C., Boamah V.E., Zumbi C.N., Osei F.B. (2018). Antibiotic Use in Poultry Production and Its Effects on Bacterial Resistance. Antimicrobial Resistance—A Global Threat.

[196] Ahmed A.M., Shimamoto T., Shimamoto T. (2013). Molecular Characterization of Multidrug-Resistant Avian Pathogenic *Escherichia coli* Isolated from Septicemic Broilers. Int. J. Med. Microbiol..

[197] Radwan I., Abd El-Halim M., Abed A. (2020). Molecular Characterization of Antimicrobial-Resistant *Escherichia coli* Isolated from Broiler Chickens. J. Vet. Med. Res..

[198] Thomrongsuwannakij T., Narinthorn R., Mahawan T., Blackall P.J. (2022). Molecular and Phenotypic Characterization of Avian Pathogenic *Escherichia coli* Isolated from Commercial Broilers and Native Chickens. Poult. Sci..

[199] Landoni M.F., Albarellos G. (2015). The Use of Antimicrobial Agents in Broiler Chickens. Vet. J..

[200] Watts A., Wigley P. (2024). Avian Pathogenic *Escherichia coli*: An Overview of Infection Biology, Antimicrobial Resistance and Vaccination. Antibiotics.

[201] Kurnia R.S., Indrawati A., Mayasari N.L.P.I., Priadi A. (2018). Molecular Detection of Genes Encoding Resistance to Tetracycline and Determination of Plasmid-Mediated Resistance to Quinolones in Avian Pathogenic *Escherichia coli* in Sukabumi, Indonesia. Vet. World.

[202] El Seedy F.R., Abed A.H., Wafaa M.M.H., Bosila A.S., Mwafy A. (2019). Antimicrobial Resistance and Molecular Characterization of Pathogenic *E. coli* Isolated from Chickens. J. Vet. Med. Res..

[203] Ibrahim R.A., Cryer T.L., Lafi S.Q., Basha E.-A., Good L., Tarazi Y.H. (2019). Identification of *Escherichia coli* from Broiler Chickens in Jordan, Their Antimicrobial Resistance, Gene Characterization and the Associated Risk Factors. BMC Vet. Res..

[204] Dhaouadi S., Soufi L., Hamza A., Fedida D., Zied C., Awadhi E., Mtibaa M., Hassen B., Cherif A., Torres C. (2020). Co-Occurrence of *mcr-1* Mediated Colistin Resistance and β-Lactamase-Encoding Genes in Multidrug-Resistant *Escherichia coli* from Broiler Chickens with Colibacillosis in Tunisia. J. Glob. Antimicrob. Resist..

[205] Partridge S.R. (2015). Resistance Mechanisms in Enterobacteriaceae. Pathology.

[206] Partridge S.R., Kwong S.M., Firth N., Jensen S.O. (2018). Mobile Genetic Elements Associated with Antimicrobial Resistance. Clin. Microbiol. Rev..

[207] Johnson T.J., Logue C.M., Johnson J.R., Kuskowski M.A., Sherwood J.S., Barnes H.J., DebRoy C., Wannemuehler Y.M., Obata-Yasuoka M., Spanjaard L. (2012). Associations between Multidrug Resistance, Plasmid Content, and Virulence Potential among Extraintestinal Pathogenic and Commensal *Escherichia coli* from Humans and Poultry. Foodborne Pathog. Dis..

[208] Tohmaz M., Askari Badouei M., Kalateh Rahmani H., Hashemi Tabar G. (2022). Antimicrobial Resistance, Virulence Associated Genes and Phylogenetic Background versus Plasmid Replicon Types: The Possible Associations in Avian Pathogenic *Escherichia coli* (APEC). BMC Vet. Res..

[209] Yoon M.Y., Kim Y.B., Ha J.S., Seo K.W., Noh E.B., Son S.H., Lee Y.J. (2020). Molecular Characteristics of Fluoroquinolone-Resistant Avian Pathogenic *Escherichia coli* Isolated from Broiler Chickens. Poult. Sci..

[210] Nógrády N., Pászti J., Pikó H., Nagy B. (2006). Class 1 Integrons and Their Conjugal Transfer with and without Virulence-Associated Genes in Extra-Intestinal and Intestinal *Escherichia coli* of Poultry. Avian Pathol..

[211] Cavicchio L., Dotto G., Giacomelli M., Giovanardi D., Grilli G., Franciosini M.P., Trocino A., Piccirillo A. (2015). Class 1 and Class 2 Integrons in Avian Pathogenic *Escherichia coli* from Poultry in Italy. Poult. Sci..

[212] Awad A., Arafat N., Elhadidy M. (2016). Genetic Elements Associated with Antimicrobial Resistance among Avian Pathogenic *Escherichia coli*. Ann. Clin. Microbiol. Antimicrob..

[213] Kalantari M., Sharifiyazdi H., Asasi K., Abdi-Hachesoo B. (2021). High Incidence of Multidrug Resistance and Class 1 and 2 Integrons in *Escherichia coli* Isolated from Broiler Chickens in South of Iran. Vet. Res. Forum..

[214] Dheilly A., Le Devendec L., Mourand G., Bouder A., Jouy E., Kempf I. (2012). Resistance Gene Transfer during Treatments for Experimental Avian Colibacillosis. Antimicrob. Agents Chemother..

[215] Oladeinde A., Cook K., Lakin S.M., Woyda R., Abdo Z., Looft T., Herrington K., Zock G., Lawrence J.P., Thomas J.C. (2019). Horizontal Gene Transfer and Acquired Antibiotic Resistance in *Salmonella Enterica* Serovar Heidelberg Following In vitro Incubation in Broiler Ceca. Appl. Environ. Microbiol..

[216] Zhang L., Levy K., Trueba G., Cevallos W., Trostle J., Foxman B., Marrs C.F., Eisenberg J.N.S. (2015). Effects of Selection Pressure and Genetic Association on the Relationship between Antibiotic Resistance and Virulence in *Escherichia coli*. Antimicrob. Agents Chemother..

[217] Abd El-Baky R.M., Ibrahim R.A., Mohamed D.S., Ahmed E.F., Hashem Z.S. (2020). Prevalence of Virulence Genes and Their Association with Antimicrobial Resistance Among Pathogenic *E. coli* Isolated from Egyptian Patients with Different Clinical Infections. Infect. Drug Resist..

[218] Dheilly A., Le Devendec L., Mourand G., Jouy E., Kempf I. (2013). Antimicrobial Resistance Selection in Avian Pathogenic *E. coli* during Treatment. Vet. Microbiol..

[219] Andersson D.I., Hughes D. (2017). Selection and Transmission of Antibiotic-Resistant Bacteria. Microbiol. Spectr..

[220] Gadde U., Kim W.H., Oh S.T., Lillehoj H.S. (2017). Alternatives to Antibiotics for Maximizing Growth Performance and Feed Efficiency in Poultry: A Review. Anim. Health Res. Rev..

[221] Lillehoj H., Liu Y., Calsamiglia S., Fernandez-Miyakawa M.E., Chi F., Cravens R.L., Oh S., Gay C.G. (2018). Phytochemicals as Antibiotic Alternatives to Promote Growth and Enhance Host Health. Vet. Res..

[222] Khameneh B., Eskin N.A.M., Iranshahy M., Fazly Bazzaz B.S. (2021). Phytochemicals: A Promising Weapon in the Arsenal against Antibiotic-Resistant Bacteria. Antibiotics.

[223] Tajkarimi M.M., Ibrahim S.A., Cliver D.O. (2010). Antimicrobial Herb and Spice Compounds in Food. Food Control.

[224] Seidavi A., Tavakoli M., Slozhenkina M., Gorlov I., Hashem N.M., Asroosh F., Taha A.E., Abd El-Hack M.E., Swelum A.A. (2021). The Use of Some Plant-Derived Products as Effective Alternatives to Antibiotic Growth Promoters in Organic Poultry Production: A Review. Environ. Sci. Pollut. Res. Int..

[225] Hotea I., Dragomirescu M., Berbecea A., Radulov I., Kamboh A.A. (2022). Phytochemicals as Alternatives to Antibiotics in Animal Production. Antibiotics and Probiotics in Animal Food—Impact and Regulation.

[226] Khare T., Anand U., Dey A., Assaraf Y.G., Chen Z.-S., Liu Z., Kumar V. (2021). Exploring Phytochemicals for Combating Antibiotic Resistance in Microbial Pathogens. Front. Pharmacol..

[227] Altemimi A., Lakhssassi N., Baharlouei A., Watson D.G., Lightfoot D.A. (2017). Phytochemicals: Extraction, Isolation, and Identification of Bioactive Compounds from Plant Extracts. Plants.

[228] Peek H.W., Halkes S.B.A., Tomassen M.M.M., Mes J.J., Landman W.J.M. (2013). In vivo Screening of Five Phytochemicals/Extracts and a Fungal Immunomodulatory Protein against Colibacillosis in Broilers. Avian Pathol..

[229] Chodkowska K.A., Iwiński H., Wódz K., Nowak T., Różański H. (2022). In vitro Assessment of Antimicrobial Activity of Phytobiotics Composition towards of Avian Pathogenic *Escherichia coli* (APEC) and Other *E. coli* Strains Isolated from Broiler Chickens. Antibiotics.

[230] Temitope O. (2015). Comparative Study of Antibacterial and Phytochemical Properties of Nigerian. Arch. Curr. Res. Int..

[231] Wagle B.R., Donoghue A.M., Jesudhasan P.R. (2021). Select Phytochemicals Reduce *Campylobacter jejuni* in Postharvest Poultry and Modulate the Virulence Attributes of *C. jejuni*. Front. Microbiol..

[232] Diaz Carrasco J.M., Redondo L.M., Redondo E.A., Dominguez J.E., Chacana A.P., Fernandez Miyakawa M.E. (2016). Use of Plant Extracts as an Effective Manner to Control *Clostridium perfringens* Induced Necrotic Enteritis in Poultry. Biomed. Res. Int..

[233] Nazir A., Malik K., Qamar H., Basit M.H., Liaqat A., Shahid M., Khan M.I., Fatima A., Irshad A., Sadia H. (2020). A Review: Use of Plant Extracts and Their Phytochemical Constituents to Control Antibiotic Resistance in *S. aureus*. Pure Appl. Biol..

[234] Awad N.F.S., Abd El-Hamid M.I., Nabil N.M., Tawakol M.M., Eid S., Al-Zaban M.I., Farouk H., Zakai S.A., Elkelish A., Ibrahim M.S. (2023). Multidrug Resistant and Multivirulent Avian Bacterial Pathogens: Tackling Experimental Leg Disorders Using Phytobiotics and Antibiotics Alone or in Combination. Poult. Sci..

[235] El-Shall N.A., Abd El-Hack M.E., Albaqami N.M., Khafaga A.F., Taha A.E., Swelum A.A., El-Saadony M.T., Salem H.M., El-Tahan A.M., AbuQamar S.F. (2022). Phytochemical Control of Poultry Coccidiosis: A Review. Poult. Sci..

[236] Kongkham B., Prabakaran D., Puttaswamy H. (2020). Opportunities and Challenges in Managing Antibiotic Resistance in Bacteria Using Plant Secondary Metabolites. Fitoterapia.

[237] Hassanzadeh K., Buccarello L., Dragotto J., Mohammadi A., Corbo M., Feligioni M. (2020). Obstacles against the Marketing of Curcumin as a Drug. Int. J. Mol. Sci..

[238] Di Lorenzo C., Colombo F., Biella S., Stockley C., Restani P. (2021). Polyphenols and Human Health: The Role of Bioavailability. Nutrients.

[239] Woyengo T.A., Nyachoti C.M. (2013). Review: Anti-Nutritional Effects of Phytic Acid in Diets for Pigs and Poultry—Current Knowledge and Directions for Future Research. Can. J. Anim. Sci..

[240] Chukwuebuka E., Chinenye I.J. (2015). Biological Functions and Anti-Nutritional Effects of Phytochemicals in Living System. J. Pharm. Biol. Sci..

[241] Abdel-Moneim A.E., Shehata A.M., Alzahrani S.O., Shafi M.E., Mesalam N.M., Taha A.E., Swelum A.A., Arif M., Fayyaz M., Abd El-Hack M.E. (2020). The Role of Polyphenols in Poultry Nutrition. J. Anim. Physiol. Anim. Nutr..

[242] Zhai H., Liu H., Wang S., Wu J., Kluenter A.-M. (2018). Potential of Essential Oils for Poultry and Pigs. Anim. Nutr..

[243] Abdelli N., Solà-Oriol D., Pérez J.F. (2021). Phytogenic Feed Additives in Poultry: Achievements, Prospective and Challenges. Animals.

[244] Wells C.W. (2024). Effects of Essential Oils on Economically Important Characteristics of Ruminant Species: A Comprehensive Review. Anim. Nutr..

[245] Yang C., Chowdhury M.A., Huo Y., Gong J. (2015). Phytogenic Compounds as Alternatives to In-Feed Antibiotics: Potentials and Challenges in Application. Pathogens.

[246] Molyneux R.J., Lee S.T., Gardner D.R., Panter K.E., James L.F. (2007). Phytochemicals: The Good, the Bad and the Ugly?. Phytochemistry.

[247] Egbuna C., Thomas S.A., Nwosu O.K., Oriyomi O.V., Kryeziu T.L., Kaliyaperumal S., Ifemeje J.C. (2018). Toxic Plants and Phytochemicals. Phytochemistry.

[248] Guldiken B., Ozkan G., Catalkaya G., Ceylan F.D., Ekin Yalcinkaya I., Capanoglu E. (2018). Phytochemicals of Herbs and Spices: Health versus Toxicological Effects. Food Chem. Toxicol..

[249] Salem M.A., Serag A., El-Seedi H.R., Hamdan D.I., Ezzat S.M., Zayed A., Mtewa A.G., Egbuna C. (2021). Identification and Analysis of Toxic Phytochemicals. Phytochemistry, the Military and Health.

[250] Hoelzer K., Bielke L., Blake D.P., Cox E., Cutting S.M., Devriendt B., Erlacher-Vindel E., Goossens E., Karaca K., Lemiere S. (2018). Vaccines as Alternatives to Antibiotics for Food Producing Animals. Part 2: New Approaches and Potential Solutions. Vet. Res..

[251] Rabie N.S., Amin Girh Z.M.S. (2020). Bacterial Vaccines in Poultry. Bull. Natl. Res. Cent..

[252] Ghunaim H., Abu-Madi M.A., Kariyawasam S. (2014). Advances in Vaccination against Avian Pathogenic *Escherichia coli* Respiratory Disease: Potentials and Limitations. Vet. Microbiol..

[253] Hajra D., Datey A., Chakravortty D. (2021). Attenuation Methods for Live Vaccines. Methods Mol. Biol..

[254] Vartak A., Sucheck S. (2016). Recent Advances in Subunit Vaccine Carriers. Vaccines.

[255] Peighambari S.M., Gyles C.L. (1998). Construction and Characterization of Avian *Escherichia coli* Cya Crp Mutants. Avian Dis..

[256] Kariyawasam S., Wilkie B.N., Gyles C.L. (2004). Construction, Characterization, and Evaluation of the Vaccine Potential of Three Genetically Defined Mutants of Avian Pathogenic *Escherichia coli*. Avian Dis..

[257] Yaguchi K., Ohgitani T., Noro T., Kaneshige T., Shimizu Y. (2009). Vaccination of Chickens with Liposomal Inactivated Avian Pathogenic *Escherichia coli* (APEC) Vaccine by Eye Drop or Coarse Spray Administration. Avian Dis..

[258] Lee J.H., Chaudhari A.A., Oh I.G., Eo S.K., Park S.-Y., Jawale C. (2015). V Immune Responses to Oral Vaccination with *Salmonella*-Delivered Avian Pathogenic *Escherichia coli* Antigens and Protective Efficacy against Colibacillosis. Can. J. Vet. Res..

[259] Koutsianos D., Gantelet H., Franzo G., Lecoupeur M., Thibault E., Cecchinato M., Koutoulis K. (2020). An Assessment of the Level of Protection Against Colibacillosis Conferred by Several Autogenous and/or Commercial Vaccination Programs in Conventional Pullets upon Experimental Challenge. Vet. Sci..

[260] Wang H., Cao L., Logue C.M., Barbieri N.L., Nolan L.K., Lin J. (2023). Evaluation of Immunogenicity and Efficacy of the Enterobactin Conjugate Vaccine in Protecting Chickens from Colibacillosis. Vaccine.

[261] Wang H., Logue C.M., Nolan L.K., Lin J. (2023). Assessment of an Enterobactin Conjugate Vaccine in Layers to Protect Their Offspring from Colibacillosis. Pathogens.

[262] Hjelm A., Söderström B., Vikström D., Jong W.S.P., Luirink J., de Gier J.-W. (2015). Autotransporter-Based Antigen Display in Bacterial Ghosts. Appl. Environ. Microbiol..

[263] Chen H., Ji H., Kong X., Lei P., Yang Q., Wu W., Jin L., Sun D. (2021). Bacterial Ghosts-Based Vaccine and Drug Delivery Systems. Pharmaceutics.

[264] Tuntufye H.N., Ons E., Pham A.D.N., Luyten T., Van Gerven N., Bleyen N., Goddeeris B.M. (2012). *Escherichia coli* Ghosts or Live *E. coli* Expressing the Ferri-Siderophore Receptors *fepA*, *fhuE*, *iroN* and *iutA* Do Not Protect Broiler Chickens against Avian Pathogenic *E. coli* (APEC). Vet. Microbiol..

[265] Ebrahimi-Nik H., Bassami M.R., Mohri M., Rad M., Khan M.I. (2018). Bacterial Ghost of Avian Pathogenic *E. coli* (APEC) Serotype O78:K80 as a Homologous Vaccine against Avian Colibacillosis. PLoS ONE.

[266] Salehi T.Z., Tabatabaei S., Karimi V., Fasaei B.N., Derakhshandeh A., Jahrom A.O.N. (2012). Assessment of Immunity against Avian Colibacillosis Induced by an AroA Mutant Containing Increased Serum Survival Gene in Broilers. Braz. J. Microbiol..

[267] El Jakee J.K., El Amry G.M., Hessain A.M., Hemeg H.A., Shafei S.M., Moussa I.M. (2016). Production and Evaluation of Autogenous Vaccine against Avian Colibacillosis. J. Anim. Plant Sci..

[268] Li L., Thøfner I., Christensen J.P., Ronco T., Pedersen K., Olsen R.H. (2017). Evaluation of the Efficacy of an Autogenous *Escherichia coli* Vaccine in Broiler Breeders. Avian Pathol..

[269] Peighambari S.M., Hunter D.B., Shewen P.E., Gyles C.L. (2002). Safety, Immunogenicity, and Efficacy of Two *Escherichia coli cya crp* Mutants as Vaccines for Broilers. Avian Dis..

[270] Vandemaele F., Ververken C., Bleyen N., Geys J., D’Hulst C., Addwebi T., van Empel P., Goddeeris B.M. (2005). Immunization with the Binding Domain of FimH, the Adhesin of Type 1 Fimbriae, Does Not Protect Chickens against Avian Pathogenic *Escherichia coli*. Avian Pathol..

[271] Vandemaele F., Bleyen N., Abuaboud O., Vandermeer E., Jacobs A., Goddeeris B.M. (2006). Immunization with the Biologically Active Lectin Domain of PapGII Induces Strong Adhesion-Inhibiting Antibody Responses but Not Protection against Avian Pathogenic *Escherichia coli*. Avian Pathol..

[272] El Sayed M.F., Soliman R.A., Ghanem H.M., Khedr M.M.S., Mohamed G.M., El Safty M.M.D. (2021). Trials for Preparation and Evaluation of a Combined Inactivated Reassorted H5N1 and *Escherichia coli* O157 Vaccine in Poultry. Vet. World.

[273] Keita A., Le Devendec L., Amelot M., Puterflam J., Lucas C., Bougeard S., Delannoy S., Schouler C., Fach P., Lucas P. (2022). Efficacy of Passive Immunization in Broiler Chicks via an Inactivated *Escherichia coli* Autogenous Vaccine Administered to Broiler Breeder Hens. Avian Pathol..

[274] Lynne A.M., Kariyawasam S., Wannemuehler Y., Johnson T.J., Johnson S.J., Sinha A.S., Lynne D.K., Moon H.W., Jordan D.M., Logue C.M. (2012). Recombinant Iss as a Potential Vaccine for Avian Colibacillosis. Avian Dis..

[275] Chaudhari A.A., Matsuda K., Lee J.H. (2013). Construction of an Attenuated *Salmonella* Delivery System Harboring Genes Encoding Various Virulence Factors of Avian Pathogenic *Escherichia coli* and Its Potential as a Candidate Vaccine for Chicken Colibacillosis. Avian Dis..

[276] La Ragione R.M., Woodward M.J., Kumar M., Rodenberg J., Fan H., Wales A.D., Karaca K. (2013). Efficacy of a Live Attenuated *Escherichia coli* O78∶K80 Vaccine in Chickens and Turkeys. Avian Dis..

[277] Ahmed H.A., Mekky H.M., El-Sadek G.M. (2012). Studies on Vaccination of Turkey Against *Escherichia coli* Infection. Glob. Vet..

[278] Nagano T., Kitahara R., Nagai S. (2012). An Attenuated Mutant of Avian Pathogenic *Escherichia coli* Serovar O78: A Possible Live Vaccine Strain for Prevention of Avian Colibacillosis. Microbiol. Immunol..

[279] Mohamed M.A., Bakhit B.M., Ibrahim A.A., Saleh M. (2016). Evaluation of The Living *Escherichia coli*-O78 Deleted AroA Vaccine Against Homologous and Heterologous *E. coli* Challenge in Broiler Chickens. J. Adv. Vet. Res..

[280] Gharib A., Hamouda A., bdel-Wahab A.A., Fawzy M. (2017). Protective Efficacy of a Commercial Live Attenuated *aroA* Mutant Vaccine Against Avian Pathogenic *Escherichia coli* Challenge in Broilers. Zagazig Vet. J..

[281] Sadeghi M., Tavakkoli H., Golchin M., Ghanbarpour R., Amanollahi S. (2018). Efficacy and Safety of Poulvac *E. coli* Vaccine in Broiler Chickens Challenged with *E. coli* Serotype O78 and an Acute Field Isolate. Comp. Clin. Path..

[282] Elbestawy A.R., Ellakany H.F., Abd El-Hamid H.S., Ibrahim M.S., Gado A.R., Mustafa N.S., Moussa I.M., Al-Maary K.S., Al-Sarar D.S., Alshammari H.O. (2021). Comparative Evaluation of a Live *E. coli* Vaccine and Cefotaxime Treatment against Three *E. coli* Serotypes in Broilers. J. King Saud Univ. Sci..

[283] Gregersen R.H., Christensen H., Ewers C., Bisgaard M. (2010). Impact of *Escherichia coli* Vaccine on Parent Stock Mortality, First Week Mortality of Broilers and Population Diversity of *E. coli* in Vaccinated Flocks. Avian Pathol..

[284] Kwaga J.K., Allan B.J., van der Hurk J.V., Seida H., Potter A.A. (1994). A *carAB* Mutant of Avian Pathogenic *Escherichia coli* Serogroup O2 Is Attenuated and Effective as a Live Oral Vaccine against Colibacillosis in Turkeys. Infect. Immun..

[285] Gyimah J.E., Panigrahy B. (1985). Immunogenicity of an *Escherichia coli* (Serotype O1) Pili Vaccine in Chickens. Avian Dis..

[286] Gyimah J.E., Panigrahy B., Williams J.D. (1986). Immunogenicity of an *Escherichia coli* Multivalent Pilus Vaccine in Chickens. Avian Dis..

[287] Lynne A.M., Foley S.L., Nolan L.K. (2006). Immune Response to Recombinant *Escherichia coli* Iss Protein in Poultry. Avian Dis..

[288] Dissanayake D.R.A., Wijewardana T.G., Gunawardena G.A., Poxton I.R. (2010). Potential Use of a Liposome-Encapsulated Mixture of Lipopolysaccharide Core Types (R1, R2, R3 and R4) of *Escherichia coli* in Controlling Colisepticaemia in Chickens. J. Med. Microbiol..

[289] Stromberg Z.R., Van Goor A., Redweik G.A.J., Mellata M. (2018). Characterization of Spleen Transcriptome and Immunity Against Avian Colibacillosis After Immunization with Recombinant Attenuated *Salmonella* Vaccine Strains. Front. Vet. Sci..

[290] Ma S.-T., Ding G.-J., Huang X.-W., Wang Z.-W., Wang L., Yu M.-L., Shi W., Jiang Y.-P., Tang L.-J., Xu Y.-G. (2018). Immunogenicity in Chickens with Orally Administered Recombinant Chicken-Borne *Lactobacillus saerimneri* Expressing FimA and OmpC Antigen of O78 Avian Pathogenic *Escherichia coli*. J. Med. Microbiol..

[291] Wittebole X., De Roock S., Opal S.M. (2014). A Historical Overview of Bacteriophage Therapy as an Alternative to Antibiotics for the Treatment of Bacterial Pathogens. Virulence.

[292] Keen E.C. (2012). Phage Therapy: Concept to Cure. Front. Microbiol..

[293] Sharma S., Chatterjee S., Datta S., Prasad R., Dubey D., Prasad R.K., Vairale M.G. (2017). Bacteriophages and Its Applications: An Overview. Folia Microbiol..

[294] Thung T.Y., Lee E., Premarathne J.M.K.J.K., Nurzafirah M., Kuan C.H., Elexson N., Tan C.W., Malcolm T.T.H., New C.Y., Ramzi O.S.B. (2018). Bacteriophages and Their Applications. Food Res..

[295] Kassa T. (2021). Bacteriophages Against Pathogenic Bacteria and Possibilities for Future Application in Africa. Infect. Drug Resist..

[296] Kutter E., De Vos D., Gvasalia G., Alavidze Z., Gogokhia L., Kuhl S., Abedon S. (2010). Phage Therapy in Clinical Practice: Treatment of Human Infections. Curr. Pharm. Biotechnol..

[297] Abedon S., Danis-Wlodarczyk K., Alves D. (2021). Phage Therapy in the 21st Century: Is There Modern, Clinical Evidence of Phage-Mediated Efficacy?. Pharmaceuticals.

[298] Ferriol-González C., Domingo-Calap P. (2021). Phage Therapy in Livestock and Companion Animals. Antibiotics.

[299] Ramos-Vivas J., Superio J., Galindo-Villegas J., Acosta F. (2021). Phage Therapy as a Focused Management Strategy in Aquaculture. Int. J. Mol. Sci..

[300] Holtappels D., Fortuna K., Lavigne R., Wagemans J. (2021). The Future of Phage Biocontrol in Integrated Plant Protection for Sustainable Crop Production. Curr. Opin. Biotechnol..

[301] Mosimann S., Desiree K., Ebner P. (2021). Efficacy of Phage Therapy in Poultry: A Systematic Review and Meta-Analysis. Poult. Sci..

[302] Huff W.E., Huff G.R., Rath N.C., Balog J.M., Donoghue A.M. (2004). Therapeutic Efficacy of Bacteriophage and Baytril (Enrofloxacin) Individually and in Combination to Treat Colibacillosis in Broilers. Poult. Sci..

[303] Huff W.E., Huff G.R., Rath N.C., Donoghue A.M. (2013). Method of Administration Affects the Ability of Bacteriophage to Prevent Colibacillosis in 1-Day-Old Broiler Chickens. Poult. Sci..

[304] Naghizadeh M., Karimi Torshizi M.A., Rahimi S., Dalgaard T.S. (2019). Synergistic Effect of Phage Therapy Using a Cocktail Rather than a Single Phage in the Control of Severe Colibacillosis in Quails. Poult. Sci..

[305] El-Gohary F.A., Huff W.E., Huff G.R., Rath N.C., Zhou Z.Y., Donoghue A.M. (2014). Environmental Augmentation with Bacteriophage Prevents Colibacillosis in Broiler Chickens. Poult. Sci..

[306] Lin J., Du F., Long M., Li P. (2022). Limitations of Phage Therapy and Corresponding Optimization Strategies: A Review. Molecules.

[307] Yen M., Cairns L.S., Camilli A. (2017). A Cocktail of Three Virulent Bacteriophages Prevents Vibrio Cholerae Infection in Animal Models. Nat. Commun..

[308] Oechslin F. (2018). Resistance Development to Bacteriophages Occurring during Bacteriophage Therapy. Viruses.

[309] Ge H., Fu S., Guo H., Hu M., Xu Z., Zhou X., Chen X., Jiao X. (2022). Application and Challenge of Bacteriophage in the Food Protection. Int. J. Food Microbiol..

[310] Labrie S.J., Samson J.E., Moineau S. (2010). Bacteriophage Resistance Mechanisms. Nat. Rev. Microbiol..

[311] Wang C., Nie T., Lin F., Connerton I.F., Lu Z., Zhou S., Hang H. (2019). Resistance Mechanisms Adopted by a *Salmonella* Typhimurium Mutant against Bacteriophage. Virus Res..

[312] Penadés J.R., Chen J., Quiles-Puchalt N., Carpena N., Novick R.P. (2015). Bacteriophage-Mediated Spread of Bacterial Virulence Genes. Curr. Opin. Microbiol..

[313] Colavecchio A., Cadieux B., Lo A., Goodridge L.D. (2017). Bacteriophages Contribute to the Spread of Antibiotic Resistance Genes among Foodborne Pathogens of the Enterobacteriaceae Family—A Review. Front. Microbiol..

[314] Schneider C.L. (2021). Bacteriophage-Mediated Horizontal Gene Transfer: Transduction. Bacteriophages.

[315] Ranjan A. (2022). The Use of Probiotics, Prebiotics, and Synbiotics as an Alternative to Antibiotics. Alternatives to Antibiotics.

[316] Mahesh M.S., Mohanta R.K., Patra A.K. (2020). Probiotics in Livestock and Poultry Nutrition and Health. Advances in Probiotics for Sustainable Food and Medicine.

[317] Zommiti M., Chikindas M.L., Ferchichi M. (2020). Probiotics—Live Biotherapeutics: A Story of Success, Limitations, and Future Prospects—Not Only for Humans. Probiotics Antimicrob. Proteins.

[318] Abd El-Hack M.E., El-Saadony M.T., Shafi M.E., Qattan S.Y.A., Batiha G.E., Khafaga A.F., Abdel-Moneim A.E., Alagawany M. (2020). Probiotics in Poultry Feed: A Comprehensive Review. J. Anim. Physiol. Anim. Nutr..

[319] Baldwin S., Hughes R.J., Hao Van T.T., Moore R.J., Stanley D. (2018). At-Hatch Administration of Probiotic to Chickens Can Introduce Beneficial Changes in Gut Microbiota. PLoS ONE.

[320] Aziz Mousavi S.M.A., Mahmoodzadeh Hosseini H., Mirhosseini S.A. (2018). A Review of Dietary Probiotics in Poultry. J. Appl. Biotechnol. Rep..

[321] Karimi A. (2022). Effect of Probiotics (Direct-Fed Microbials) in Poultry Production: A Comprehensive Review. Rev. Electron. Vet..

[322] Wolfenden R.E., Pumford N.R., Morgan M.J., Shivaramaiah S., Wolfenden A.D., Pixley C.M., Green J., Tellez G., Hargis B.M. (2011). Evaluation of Selected Direct-Fed Microbial Candidates on Live Performance and *Salmonella* Reduction in Commercial Turkey Brooding Houses. Poult. Sci..

[323] Shivaramaiah S., Pumford N.R., Morgan M.J., Wolfenden R.E., Wolfenden A.D., Torres-Rodríguez A., Hargis B.M., Téllez G. (2011). Evaluation of *Bacillus* Species as Potential Candidates for Direct-Fed Microbials in Commercial Poultry. Poult. Sci..

[324] Latorre J.D., Hernandez-Velasco X., Bielke L.R., Vicente J.L., Wolfenden R., Menconi A., Hargis B.M., Tellez G. (2015). Evaluation of a *Bacillus* Direct-Fed Microbial Candidate on Digesta Viscosity, Bacterial Translocation, Microbiota Composition and Bone Mineralisation in Broiler Chickens Fed on a Rye-Based Diet. Br. Poult. Sci..

[325] Tarabees R., El-Sayed M.S., Shehata A.A., Diab M.S. (2020). Effects of the Probiotic Candidate *E. faecalis*-1, the Poulvac *E. coli* Vaccine, and Their Combination on Growth Performance, Caecal Microbial Composition, Immune Response, and Protection against *E. coli* O78 Challenge in Broiler Chickens. Probiotics Antimicrob. Proteins.

[326] Tarabees R., Gafar K.M., EL-Sayed M.S., Shehata A.A., Ahmed M. (2019). Effects of Dietary Supplementation of Probiotic Mix and Prebiotic on Growth Performance, Cecal Microbiota Composition, and Protection Against *Escherichia coli* O78 in Broiler Chickens. Probiotics Antimicrob. Proteins.

[327] Hofacre C.L., Johnson A.C., Kelly B.J., Froyman R. (2002). Effect of a Commercial Competitive Exclusion Culture on Reduction of Colonization of an Antibiotic-Resistant Pathogenic *Escherichia coli* in Day-Old Broiler Chickens. Avian Dis..

[328] Ceccarelli D., van Essen-Zandbergen A., Smid B., Veldman K.T., Boender G.J., Fischer E.A.J., Mevius D.J., van der Goot J.A. (2017). Competitive Exclusion Reduces Transmission and Excretion of Extended-Spectrum-β-Lactamase-Producing *Escherichia coli* in Broilers. Appl. Environ. Microbiol..

[329] Ebrahem A.F., El-Demerdash A.S., Orady R.M., Nabil N.M. (2024). Modulatory Effect of Competitive Exclusion on the Transmission of ESBL *E. coli* in Chickens. Probiotics Antimicrob. Proteins.

[330] Li T., Teng D., Mao R., Hao Y., Wang X., Wang J. (2020). A Critical Review of Antibiotic Resistance in Probiotic Bacteria. Food Res. Int..

[331] Amachawadi R.G., Giok F., Shi X., Soto J., Narayanan S.K., Tokach M.D., Apley M.D., Nagaraja T.G. (2018). Antimicrobial Resistance of *Enterococcus Faecium* Strains Isolated from Commercial Probiotic Products Used in Cattle and Swine1,2. J. Anim. Sci..

[332] Chantziaras I., Smet A., Filippitzi M.E., Damiaans B., Haesebrouck F., Boyen F., Dewulf J. (2018). The Effect of a Commercial Competitive Exclusion Product on the Selection of Enrofloxacin Resistance in Commensal *E. coli* in Broilers. Avian Pathol..

[333] Fu S., Yang Q., He F., Lan R., Hao J., Ni P., Liu Y., Li R. (2020). National Safety Survey of Animal-Use Commercial Probiotics and Their Spillover Effects from Farm to Humans: An Emerging Threat to Public Health. Clin. Infect. Dis..

[334] Wright A. (2005). Regulating the Safety of Probiotics—The European Approach. Curr. Pharm. Des..

[335] Liang D., Wu F., Zhou D., Tan B., Chen T. (2024). Commercial Probiotic Products in Public Health: Current Status and Potential Limitations. Crit. Rev. Food Sci. Nutr..

[336] Findik F. (2021). Nanomaterials and Their Applications. Period. Eng. Nat. Sci. (PEN).

[337] Kumari S., Sarkar L. (2021). A Review on Nanoparticles: Structure, Classification, Synthesis & Applications. J. Sci. Res..

[338] Altammar K.A. (2023). A Review on Nanoparticles: Characteristics, Synthesis, Applications, and Challenges. Front. Microbiol..

[339] Marinescu L., Ficai D., Oprea O., Marin A., Ficai A., Andronescu E., Holban A.-M. (2020). Optimized Synthesis Approaches of Metal Nanoparticles with Antimicrobial Applications. J. Nanomater..

[340] Vassallo A., Silletti M.F., Faraone I., Milella L. (2020). Nanoparticulate Antibiotic Systems as Antibacterial Agents and Antibiotic Delivery Platforms to Fight Infections. J. Nanomater..

[341] Hosseini S.M., Taheri M., Nouri F., Farmani A., Moez N.M., Arabestani M.R. (2022). Nano Drug Delivery in Intracellular Bacterial Infection Treatments. Biomed. Pharmacother..

[342] Naskar A., Kim K. (2019). Nanomaterials as Delivery Vehicles and Components of New Strategies to Combat Bacterial Infections: Advantages and Limitations. Microorganisms.

[343] Zhuo Y., Zhao Y.-G., Zhang Y. (2024). Enhancing Drug Solubility, Bioavailability, and Targeted Therapeutic Applications through Magnetic Nanoparticles. Molecules.

[344] Hussain S., Joo J., Kang J., Kim B., Braun G.B., She Z.-G., Kim D., Mann A.P., Mölder T., Teesalu T. (2018). Antibiotic-Loaded Nanoparticles Targeted to the Site of Infection Enhance Antibacterial Efficacy. Nat. Biomed. Eng..

[345] Cerbu C., Kah M., White J.C., Astete C.E., Sabliov C.M. (2021). Fate of Biodegradable Engineered Nanoparticles Used in Veterinary Medicine as Delivery Systems from a One Health Perspective. Molecules.

[346] Nisar P., Ali N., Rahman L., Ali M., Shinwari Z.K. (2019). Antimicrobial Activities of Biologically Synthesized Metal Nanoparticles: An Insight into the Mechanism of Action. J. Biol. Inorg. Chem..

[347] Brandelli A., Ritter A.C., Veras F.F. (2017). Antimicrobial Activities of Metal Nanoparticles. Metal Nanoparticles in Pharma.

[348] Dakal T.C., Kumar A., Majumdar R.S., Yadav V. (2016). Mechanistic Basis of Antimicrobial Actions of Silver Nanoparticles. Front. Microbiol..

[349] Cheon J.Y., Kim S.J., Rhee Y.H., Kwon O.H., Park W.H. (2019). Shape-Dependent Antimicrobial Activities of Silver Nanoparticles. Int. J. Nanomed..

[350] Sayed F.A.-Z., Eissa N.G., Shen Y., Hunstad D.A., Wooley K.L., Elsabahy M. (2022). Morphologic Design of Nanostructures for Enhanced Antimicrobial Activity. J. Nanobiotechnol..

[351] Goyal D., Kaur G., Tewari R., Kumar R. (2017). Correlation of Edge Truncation with Antibacterial Activity of Plate-like Anisotropic Silver Nanoparticles. Environ. Sci. Pollut. Res..

[352] Kaur H., Rauwel P., Rauwel E. (2023). Antimicrobial Nanoparticles: Synthesis, Mechanism of Actions. Antimicrobial Activity of Nanoparticles.

[353] Slavin Y.N., Asnis J., Häfeli U.O., Bach H. (2017). Metal Nanoparticles: Understanding the Mechanisms behind Antibacterial Activity. J. Nanobiotechnol..

[354] Wang L., Hu C., Shao L. (2017). The Antimicrobial Activity of Nanoparticles: Present Situation and Prospects for the Future. Int. J. Nanomedicine.

[355] Lam P.-L., Wong R.S.-M., Lam K.-H., Hung L.-K., Wong M.-M., Yung L.-H., Ho Y.-W., Wong W.-Y., Hau D.K.-P., Gambari R. (2020). The Role of Reactive Oxygen Species in the Biological Activity of Antimicrobial Agents: An Updated Mini Review. Chem. Biol. Interact..

[356] Duffy L.L., Osmond-McLeod M.J., Judy J., King T. (2018). Investigation into the Antibacterial Activity of Silver, Zinc Oxide and Copper Oxide Nanoparticles against Poultry-Relevant Isolates of *Salmonella* and *Campylobacter*. Food Control.

[357] Gabrielyan L., Badalyan H., Gevorgyan V., Trchounian A. (2020). Comparable Antibacterial Effects and Action Mechanisms of Silver and Iron Oxide Nanoparticles on *Escherichia coli* and *Salmonella* Typhimurium. Sci. Rep..

[358] Abdalhamed A.M., Ghazy A.A., Ibrahim E.S., Arafa A.A., Zeedan G.S.G. (2021). Therapeutic Effect of Biosynthetic Gold Nanoparticles on Multidrug-Resistant *Escherichia coli* and *Salmonella* Species Isolated from Ruminants. Vet. World.

[359] Sharma R.P., Raut S.D., Jadhav V.V., Mulani R.M., Kadam A.S., Mane R.S. (2022). Assessment of Antibacterial and Anti-Biofilm Effects of Zinc Ferrite Nanoparticles against *Klebsiella pneumoniae*. Folia Microbiol..

[360] Hassan P.B., Ameen S.S.M., Mohammed L., Ameen S.M.M., Omer K.M. (2024). Enhanced Antibacterial Activity of a Novel Silver-Based Metal Organic Framework towards Multidrug-Resistant *Klebsiella pneumonia*. Nanoscale Adv..

[361] Jesline A., John N.P., Narayanan P.M., Vani C., Murugan S. (2015). Antimicrobial Activity of Zinc and Titanium Dioxide Nanoparticles against Biofilm-Producing Methicillin-Resistant *Staphylococcus aureus*. Appl. Nanosci..

[362] Ahmed H.A., El Bayomi R.M., Hamed R.I., Mohsen R.A., El-Gohary F.A., Hefny A.A., Elkhawaga E., Tolba H.M.N. (2022). Genetic Relatedness, Antibiotic Resistance, and Effect of Silver Nanoparticle on Biofilm Formation by *Clostridium perfringens* Isolated from Chickens, Pigeons, Camels, and Human Consumers. Vet. Sci..

[363] Attallah N.G.M., Elekhnawy E., Negm W.A., Hussein I.A., Mokhtar F.A., Al-Fakhrany O.M. (2022). In vivo and In vitro Antimicrobial Activity of Biogenic Silver Nanoparticles against *Staphylococcus aureus* Clinical Isolates. Pharmaceuticals.

[364] Bankier C., Matharu R.K., Cheong Y.K., Ren G.G., Cloutman-Green E., Ciric L. (2019). Synergistic Antibacterial Effects of Metallic Nanoparticle Combinations. Sci. Rep..

[365] Al-Edhari B., Mashreghi M., Makhdoumi A., Darroudi M. (2021). Antibacterial and Antibiofilm Efficacy of Ag NPs, Ni NPs and Al_2_O_3_ NPs Singly and in Combination against Multidrug-Resistant Klebsiella Pneumoniae Isolates. J. Trace Elem. Med. Biol..

[366] Panáček A., Kvítek L., Smékalová M., Večeřová R., Kolář M., Röderová M., Dyčka F., Šebela M., Prucek R., Tomanec O. (2018). Bacterial Resistance to Silver Nanoparticles and How to Overcome It. Nat. Nanotechnol..

[367] Kamat S., Kumari M. (2023). Emergence of Microbial Resistance against Nanoparticles: Mechanisms and Strategies. Front. Microbiol..

[368] Niño-Martínez N., Salas Orozco M.F., Martínez-Castañón G.-A., Torres Méndez F., Ruiz F. (2019). Molecular Mechanisms of Bacterial Resistance to Metal and Metal Oxide Nanoparticles. Int. J. Mol. Sci..

[369] Liu N., Tang M. (2020). Toxic Effects and Involved Molecular Pathways of Nanoparticles on Cells and Subcellular Organelles. J. Appl. Toxicol..

[370] Yu Z., Li Q., Wang J., Yu Y., Wang Y., Zhou Q., Li P. (2020). Reactive Oxygen Species-Related Nanoparticle Toxicity in the Biomedical Field. Nanoscale Res. Lett..

[371] Ameh T., Gibb M., Stevens D., Pradhan S.H., Braswell E., Sayes C.M. (2022). Silver and Copper Nanoparticles Induce Oxidative Stress in Bacteria and Mammalian Cells. Nanomaterials.

[372] Utembe W., Tlotleng N., Kamng’ona A. (2022). A Systematic Review on the Effects of Nanomaterials on Gut Microbiota. Curr. Res. Microb. Sci..

[373] Ma Y., Zhang J., Yu N., Shi J., Zhang Y., Chen Z., Jia G. (2023). Effect of Nanomaterials on Gut Microbiota. Toxics.

[374] Arora R., Roy T., Adak P. (2024). A Review of The Impact of Nanoparticles on Environmental Processes. BIO Web Conf..

[375] Ray P.C., Yu H., Fu P.P. (2009). Toxicity and Environmental Risks of Nanomaterials: Challenges and Future Needs. J. Environ. Sci. Health C.

[376] Bundschuh M., Filser J., Lüderwald S., McKee M.S., Metreveli G., Schaumann G.E., Schulz R., Wagner S. (2018). Nanoparticles in the Environment: Where Do We Come from, Where Do We Go To?. Environ. Sci. Eur..

[377] Heselpoth R.D., Swift S.M., Linden S.B., Mitchell M.S., Nelson D.C. (2021). Enzybiotics: Endolysins and Bacteriocins. Bacteriophages.

[378] Danis-Wlodarczyk K.M., Wozniak D.J., Abedon S.T. (2021). Treating Bacterial Infections with Bacteriophage-Based Enzybiotics: In vitro, In vivo and Clinical Application. Antibiotics.

[379] Oliveira H., Melo L.D.R., Santos S.B., Capelo-Martínez J.L., Igrejas G. (2019). Bacteriophage Proteins as Antimicrobials to Combat Antibiotic Resistance. Antibiotic Drug Resistance.

[380] Fernandes S., São-José C. (2018). Enzymes and Mechanisms Employed by Tailed Bacteriophages to Breach the Bacterial Cell Barriers. Viruses.

[381] Chang R.Y.K., Nang S.C., Chan H.-K., Li J. (2022). Novel Antimicrobial Agents for Combating Antibiotic-Resistant Bacteria. Adv. Drug Deliv. Rev..

[382] Rahman M.U., Wang W., Sun Q., Shah J.A., Li C., Sun Y., Li Y., Zhang B., Chen W., Wang S. (2021). Endolysin, a Promising Solution against Antimicrobial Resistance. Antibiotics.

[383] Aslam B., Arshad M.I., Aslam M.A., Muzammil S., Siddique A.B., Yasmeen N., Khurshid M., Rasool M., Ahmad M., Rasool M.H. (2021). Bacteriophage Proteome: Insights and Potentials of an Alternate to Antibiotics. Infect. Dis. Ther..

[384] Khan F.M., Rasheed F., Yang Y., Liu B., Zhang R. (2024). Endolysins: A New Antimicrobial Agent against Antimicrobial Resistance. Strategies and Opportunities in Overcoming the Challenges of Endolysins against Gram-Negative Bacteria. Front. Pharmacol..

[385] Abdelrahman F., Easwaran M., Daramola O.I., Ragab S., Lynch S., Oduselu T.J., Khan F.M., Ayobami A., Adnan F., Torrents E. (2021). Phage-Encoded Endolysins. Antibiotics.

[386] Shemyakin I.G., Firstova V.V., Fursova N.K., Abaev I.V., Filippovich S.Y., Ignatov S.G., Dyatlov I.A. (2020). Next-Generation Antibiotics, Bacteriophage Endolysins, and Nanomaterials for Combating Pathogens. Biochemistry.

[387] Sabur A., Khan A., Borphukan B., Razzak A., Salimullah M., Khatun M. (2025). The Unique Capability of Endolysin to Tackle Antibiotic Resistance: Cracking the Barrier. J. Xenobiot..

[388] Ho M.K.Y., Zhang P., Chen X., Xia J., Leung S.S.Y. (2022). Bacteriophage Endolysins against Gram-Positive Bacteria, an Overview on the Clinical Development and Recent Advances on the Delivery and Formulation Strategies. Crit. Rev. Microbiol..

[389] Jeong T.-H., Hong H.-W., Kim M.S., Song M., Myung H. (2023). Characterization of Three Different Endolysins Effective against Gram-Negative Bacteria. Viruses.

[390] Liu H., Hu Z., Li M., Yang Y., Lu S., Rao X. (2023). Therapeutic Potential of Bacteriophage Endolysins for Infections Caused by Gram-Positive Bacteria. J. Biomed. Sci..

[391] Mondal S.I., Draper L.A., Ross R.P., Hill C. (2020). Bacteriophage Endolysins as a Potential Weapon to Combat *Clostridioides difficile* Infection. Gut Microbes.

[392] Gontijo M.T.P., Jorge G.P., Brocchi M. (2021). Current Status of Endolysin-Based Treatments against Gram-Negative Bacteria. Antibiotics.

[393] Ajuebor J., McAuliffe O., O’Mahony J., Ross R.P., Hill C., Coffey A. (2016). Bacteriophage Endolysins and Their Applications. Sci. Prog..

[394] Golban M., Charostad J., Kazemian H., Heidari H. (2025). Phage-Derived Endolysins Against Resistant *Staphylococcus* spp.: A Review of Features, Antibacterial Activities, and Recent Applications. Infect. Dis. Ther..

[395] Lai W.C.B., Chen X., Ho M.K.Y., Xia J., Leung S.S.Y. (2020). Bacteriophage-Derived Endolysins to Target Gram-Negative Bacteria. Int. J. Pharm..

[396] Sisson H.M., Jackson S.A., Fagerlund R.D., Warring S.L., Fineran P.C. (2024). Gram-Negative Endolysins: Overcoming the Outer Membrane Obstacle. Curr. Opin. Microbiol..

[397] Bai J., Lee S., Ryu S. (2020). Identification and in vitro Characterization of a Novel Phage Endolysin That Targets Gram-Negative Bacteria. Microorganisms.

[398] Nazir A., Xu X., Liu Y., Chen Y. (2023). Phage Endolysins: Advances in the World of Food Safety. Cells.

[399] Zermeño-Cervantes L.A., Martínez-Díaz S.F., Venancio-Landeros A.A., Cardona-Félix C.S. (2023). Evaluating the Efficacy of Endolysins and Membrane Permeabilizers against Vibrio Parahaemolyticus in Marine Conditions. Res. Microbiol..

[400] Yang H., Yu J., Wei H. (2014). Engineered Bacteriophage Lysins as Novel Anti-Infectives. Front. Microbiol..

[401] Carratalá J.V., Arís A., Garcia-Fruitós E., Ferrer-Miralles N. (2023). Design Strategies for Positively Charged Endolysins: Insights into Artilysin Development. Biotechnol. Adv..

[402] Briers Y., Walmagh M., Van Puyenbroeck V., Cornelissen A., Cenens W., Aertsen A., Oliveira H., Azeredo J., Verween G., Pirnay J.-P. (2014). Engineered Endolysin-Based “Artilysins” To Combat Multidrug-Resistant Gram-Negative Pathogens. mBio.

[403] Son S.M., Kim J., Ryu S. (2023). Development of Sensitizer Peptide-Fused Endolysin Lys1S-L9P Acting against Multidrug-Resistant Gram-Negative Bacteria. Front. Microbiol..

[404] Zheng T., Zhang C. (2024). Engineering Strategies and Challenges of Endolysin as an Antibacterial Agent against Gram-negative Bacteria. Microb. Biotechnol..

[405] Röhrig C., Huemer M., Lorgé D., Luterbacher S., Phothaworn P., Schefer C., Sobieraj A.M., Zinsli L.V., Mairpady Shambat S., Leimer N. (2020). Targeting Hidden Pathogens: Cell-Penetrating Enzybiotics Eradicate Intracellular Drug-Resistant *Staphylococcus aureus*. mBio.

[406] Vander Elst N., Bert J., Favoreel H., Lavigne R., Meyer E., Briers Y. (2023). Development of Engineered Endolysins with in vitro Intracellular Activity against Streptococcal Bovine Mastitis-causing Pathogens. Microb. Biotechnol..

[407] Jun S.Y., Jung G.M., Yoon S.J., Choi Y.-J., Koh W.S., Moon K.S., Kang S.H. (2014). Preclinical Safety Evaluation of Intravenously Administered SAL200 Containing the Recombinant Phage Endolysin SAL-1 as a Pharmaceutical Ingredient. Antimicrob. Agents Chemother..

[408] Harhala M., Nelson D.C., Miernikiewicz P., Heselpoth R.D., Brzezicka B., Majewska J., Linden S.B., Shang X., Szymczak A., Lecion D. (2018). Safety Studies of Pneumococcal Endolysins Cpl-1 and Pal. Viruses.

[409] Antonova N.P., Vasina D.V., Grigoriev I.V., Laishevtsev A.I., Kapustin A.V., Savinov V.A., Vorobev A.M., Aleshkin A.V., Zackharova A.A., Remizov T.A. (2024). Pharmacokinetic and Preclinical Safety Studies of Endolysin-Based Therapeutic for Intravenous Administration. Int. J. Antimicrob. Agents.

[410] Schmelcher M., Loessner M.J. (2021). Bacteriophage Endolysins—Extending Their Application to Tissues and the Bloodstream. Curr. Opin. Biotechnol..

[411] Seijsing J., Sobieraj A.M., Keller N., Shen Y., Zinkernagel A.S., Loessner M.J., Schmelcher M. (2018). Improved Biodistribution and Extended Serum Half-Life of a Bacteriophage Endolysin by Albumin Binding Domain Fusion. Front. Microbiol..

[412] Wang Y., Wang X., Liu X., Lin B. (2024). Research Progress on Strategies for Improving the Enzyme Properties of Bacteriophage Endolysins. J. Microbiol. Biotechnol..

[413] Horvath P., Barrangou R. (2010). CRISPR/Cas, the Immune System of Bacteria and Archaea. Science.

[414] Barrangou R. (2015). The Roles of CRISPR–Cas Systems in Adaptive Immunity and Beyond. Curr. Opin. Immunol..

[415] Grissa I., Vergnaud G., Pourcel C. (2007). The CRISPRdb Database and Tools to Display CRISPRs and to Generate Dictionaries of Spacers and Repeats. BMC Bioinform..

[416] Karimi Z., Ahmadi A., Najafi A., Ranjbar R. (2018). Bacterial CRISPR Regions: General Features and Their Potential for Epidemiological Molecular Typing Studies. Open Microbiol. J..

[417] Hille F., Charpentier E. (2016). CRISPR-Cas: Biology, Mechanisms and Relevance. Philos. Trans. R. Soc. B Biol. Sci..

[418] Strich J.R., Chertow D.S. (2019). CRISPR-Cas Biology and Its Application to Infectious Diseases. J. Clin. Microbiol..

[419] Mohanraju P., Makarova K.S., Zetsche B., Zhang F., Koonin E.V., van der Oost J. (2016). Diverse Evolutionary Roots and Mechanistic Variations of the CRISPR-Cas Systems. Science.

[420] Makarova K.S., Wolf Y.I., Iranzo J., Shmakov S.A., Alkhnbashi O.S., Brouns S.J.J., Charpentier E., Cheng D., Haft D.H., Horvath P. (2020). Evolutionary Classification of CRISPR–Cas Systems: A Burst of Class 2 and Derived Variants. Nat. Rev. Microbiol..

[421] Koonin E.V., Makarova K.S. (2019). Origins and Evolution of CRISPR-Cas Systems. Philos. Trans. R. Soc. B Biol. Sci.

[422] Shmakov S., Smargon A., Scott D., Cox D., Pyzocha N., Yan W., Abudayyeh O.O., Gootenberg J.S., Makarova K.S., Wolf Y.I. (2017). Diversity and Evolution of Class 2 CRISPR–Cas Systems. Nat. Rev. Microbiol..

[423] Makarova K.S., Haft D.H., Barrangou R., Brouns S.J.J., Charpentier E., Horvath P., Moineau S., Mojica F.J.M., Wolf Y.I., Yakunin A.F. (2011). Evolution and Classification of the CRISPR–Cas Systems. Nat. Rev. Microbiol..

[424] Cui L., Bikard D. (2016). Consequences of Cas9 Cleavage in the Chromosome of *Escherichia coli*. Nucleic Acids Res..

[425] Gleditzsch D., Pausch P., Müller-Esparza H., Özcan A., Guo X., Bange G., Randau L. (2019). PAM Identification by CRISPR-Cas Effector Complexes: Diversified Mechanisms and Structures. RNA Biol..

[426] Gomaa A.A., Klumpe H.E., Luo M.L., Selle K., Barrangou R., Beisel C.L. (2014). Programmable Removal of Bacterial Strains by Use of Genome-Targeting CRISPR-Cas Systems. mBio.

[427] Rodrigues M., McBride S.W., Hullahalli K., Palmer K.L., Duerkop B.A. (2019). Conjugative Delivery of CRISPR-Cas9 for the Selective Depletion of Antibiotic-Resistant Enterococci. Antimicrob. Agents Chemother..

[428] Hamilton T.A., Pellegrino G.M., Therrien J.A., Ham D.T., Bartlett P.C., Karas B.J., Gloor G.B., Edgell D.R. (2019). Efficient Inter-Species Conjugative Transfer of a CRISPR Nuclease for Targeted Bacterial Killing. Nat. Commun..

[429] Bikard D., Euler C.W., Jiang W., Nussenzweig P.M., Goldberg G.W., Duportet X., Fischetti V.A., Marraffini L.A. (2014). Exploiting CRISPR-Cas Nucleases to Produce Sequence-Specific Antimicrobials. Nat. Biotechnol..

[430] Park J.Y., Moon B.Y., Park J.W., Thornton J.A., Park Y.H., Seo K.S. (2017). Genetic Engineering of a Temperate Phage-Based Delivery System for CRISPR/Cas9 Antimicrobials against *Staphylococcus aureus*. Sci. Rep..

[431] Mayorga-Ramos A., Zúñiga-Miranda J., Carrera-Pacheco S.E., Barba-Ostria C., Guamán L.P. (2023). CRISPR-Cas-Based Antimicrobials: Design, Challenges, and Bacterial Mechanisms of Resistance. ACS Infect. Dis..

[432] Wang X., Lyu Y., Wang S., Zheng Q., Feng E., Zhu L., Pan C., Wang S., Wang D., Liu X. (2021). Application of CRISPR/Cas9 System for Plasmid Elimination and Bacterial Killing of *Bacillus cereus* Group Strains. Front. Microbiol..

[433] Kiga K., Tan X.-E., Ibarra-Chávez R., Watanabe S., Aiba Y., Sato’o Y., Li F.-Y., Sasahara T., Cui B., Kawauchi M. (2020). Development of CRISPR-Cas13a-Based Antimicrobials Capable of Sequence-Specific Killing of Target Bacteria. Nat. Commun..

[434] Kundar R., Gokarn K. (2022). CRISPR-Cas System: A Tool to Eliminate Drug-Resistant Gram-Negative Bacteria. Pharmaceuticals.

[435] Jothi R., Karthika C., Kamaladevi A., Satish L., Pandian S.K., Gowrishankar S., Singh V. (2021). CRISPR Based Bacterial Genome Editing and Removal of Pathogens. Reprogramming the Genome: Applications of CRISPR-Cas in Non-Mammalian Systems Part A.

[436] Palacios Araya D., Palmer K.L., Duerkop B.A. (2021). CRISPR-Based Antimicrobials to Obstruct Antibiotic-Resistant and Pathogenic Bacteria. PLoS Pathog..

[437] Wang P., He D., Li B., Guo Y., Wang W., Luo X., Zhao X., Wang X. (2019). Eliminating Mcr-1-Harbouring Plasmids in Clinical Isolates Using the CRISPR/Cas9 System. J. Antimicrob. Chemother..

[438] Li P., Wan P., Zhao R., Chen J., Li X., Li J., Xiong W., Zeng Z. (2022). Targeted Elimination of *bla*_NDM-5_ Gene in *Escherichia coli* by Conjugative CRISPR-Cas9 System. Infect. Drug Resist..

[439] Tao S., Hu C., Fang Y., Zhang H., Xu Y., Zheng L., Chen L., Liang W. (2023). Targeted Elimination of Vancomycin Resistance Gene VanA by CRISPR-Cas9 System. BMC Microbiol..

[440] Buckner M.M.C., Ciusa M.L., Piddock L.J. (2018). V Strategies to Combat Antimicrobial Resistance: Anti-Plasmid and Plasmid Curing. FEMS Microbiol. Rev..

[441] Reid C.J., Cummins M.L., Börjesson S., Brouwer M.S.M., Hasman H., Hammerum A.M., Roer L., Hess S., Berendonk T., Nešporová K. (2022). A Role for ColV Plasmids in the Evolution of Pathogenic *Escherichia coli* ST58. Nat. Commun..

[442] Citorik R.J., Mimee M., Lu T.K. (2014). Sequence-Specific Antimicrobials Using Efficiently Delivered RNA-Guided Nucleases. Nat. Biotechnol..

[443] López-Igual R., Bernal-Bayard J., Rodríguez-Patón A., Ghigo J.-M., Mazel D. (2019). Engineered Toxin–Intein Antimicrobials Can Selectively Target and Kill Antibiotic-Resistant Bacteria in Mixed Populations. Nat. Biotechnol..

[444] Dorado-Morales P., Lambérioux M., Mazel D. (2024). Unlocking the Potential of Microbiome Editing: A Review of Conjugation-based Delivery. Mol. Microbiol..

[445] Neil K., Allard N., Grenier F., Burrus V., Rodrigue S. (2020). Highly Efficient Gene Transfer in the Mouse Gut Microbiota Is Enabled by the Incl2 Conjugative Plasmid TP114. Commun. Biol..

[446] Neil K., Allard N., Roy P., Grenier F., Menendez A., Burrus V., Rodrigue S. (2021). High-efficiency Delivery of CRISPR-Cas9 by Engineered Probiotics Enables Precise Microbiome Editing. Mol. Syst. Biol..

[447] Gholizadeh P., Köse Ş., Dao S., Ganbarov K., Tanomand A., Dal T., Aghazadeh M., Ghotaslou R., Ahangarzadeh Rezaee M., Yousefi B. (2020). How CRISPR-Cas System Could Be Used to Combat Antimicrobial Resistance. Infect. Drug Resist..

[448] Jain A., Srivastava P. (2013). Broad Host Range Plasmids. FEMS Microbiol. Lett..

[449] Fage C., Lemire N., Moineau S. (2021). Delivery of CRISPR-Cas Systems Using Phage-Based Vectors. Curr. Opin. Biotechnol..

[450] Yosef I., Goren M.G., Globus R., Molshanski-Mor S., Qimron U. (2017). Extending the Host Range of Bacteriophage Particles for DNA Transduction. Mol. Cell.

[451] Gencay Y.E., Jasinskytė D., Robert C., Semsey S., Martínez V., Petersen A.Ø., Brunner K., de Santiago Torio A., Salazar A., Turcu I.C. (2024). Engineered Phage with Antibacterial CRISPR–Cas Selectively Reduce *E. coli* Burden in Mice. Nat. Biotechnol..

[452] Zhang X.-H., Tee L.Y., Wang X.-G., Huang Q.-S., Yang S.-H. (2015). Off-Target Effects in CRISPR/Cas9-Mediated Genome Engineering. Mol. Ther. Nucleic Acids.

[453] Pacesa M., Lin C.-H., Cléry A., Saha A., Arantes P.R., Bargsten K., Irby M.J., Allain F.H.-T., Palermo G., Cameron P. (2022). Structural Basis for Cas9 Off-Target Activity. Cell.

[454] Anderson E.M., Haupt A., Schiel J.A., Chou E., Machado H.B., Strezoska Ž., Lenger S., McClelland S., Birmingham A., Vermeulen A. (2015). Systematic Analysis of CRISPR–Cas9 Mismatch Tolerance Reveals Low Levels of off-Target Activity. J. Biotechnol..

[455] Javed M.U., Hayat M.T., Mukhtar H., Imre K. (2023). CRISPR-Cas9 System: A Prospective Pathway toward Combatting Antibiotic Resistance. Antibiotics.

[456] Ishida K., Gee P., Hotta A. (2015). Minimizing Off-Target Mutagenesis Risks Caused by Programmable Nucleases. Int. J. Mol. Sci..

[457] Guo C., Ma X., Gao F., Guo Y. (2023). Off-Target Effects in CRISPR/Cas9 Gene Editing. Front. Bioeng. Biotechnol..

[458] Asmamaw Mengstie M., Teshome Azezew M., Asmamaw Dejenie T., Teshome A.A., Tadele Admasu F., Behaile Teklemariam A., Tilahun Mulu A., Mekonnen Agidew M., Adugna D.G., Geremew H. (2024). Recent Advancements in Reducing the Off-Target Effect of CRISPR-Cas9 Genome Editing. Biologics.

[459] Uribe R.V., Rathmer C., Jahn L.J., Ellabaan M.M.H., Li S.S., Sommer M.O.A. (2021). Bacterial Resistance to CRISPR-Cas Antimicrobials. Sci. Rep..

[460] Pursey E., Sünderhauf D., Gaze W.H., Westra E.R., van Houte S. (2018). CRISPR-Cas Antimicrobials: Challenges and Future Prospects. PLoS Pathog..

[461] Davidson A.R., Lu W.-T., Stanley S.Y., Wang J., Mejdani M., Trost C.N., Hicks B.T., Lee J., Sontheimer E.J. (2020). Anti-CRISPRs: Protein Inhibitors of CRISPR-Cas Systems. Annu. Rev. Biochem..

[462] Zerbini F., Zanella I., Fraccascia D., König E., Irene C., Frattini L.F., Tomasi M., Fantappiè L., Ganfini L., Caproni E. (2017). Large Scale Validation of an Efficient CRISPR/Cas-Based Multi Gene Editing Protocol in *Escherichia coli*. Microb. Cell Fact..

[463] Lam K.N., Spanogiannopoulos P., Soto-Perez P., Alexander M., Nalley M.J., Bisanz J.E., Nayak R.R., Weakley A.M., Yu F.B., Turnbaugh P.J. (2021). Phage-Delivered CRISPR-Cas9 for Strain-Specific Depletion and Genomic Deletions in the Gut Microbiome. Cell Rep..

[464] Fischer S., Maier L.-K., Stoll B., Brendel J., Fischer E., Pfeiffer F., Dyall-Smith M., Marchfelder A. (2012). An Archaeal Immune System Can Detect Multiple Protospacer Adjacent Motifs (PAMs) to Target Invader DNA. J. Biol. Chem..

[465] Huang J., Ding K., Chen J., Fan J., Huang L., Qiu S., Wang L., Du X., Wang C., Pan H. (2025). Comparison of CRISPR-Cas9, CRISPR-Cas12f1, and CRISPR-Cas3 in Eradicating Resistance Genes KPC-2 and IMP-4. Microbiol. Spectr..

[466] Hullahalli K., Rodrigues M., Palmer K.L. (2017). Exploiting CRISPR-Cas to Manipulate *Enterococcus faecalis* Populations. eLife.

[467] Qi L.S., Larson M.H., Gilbert L.A., Doudna J.A., Weissman J.S., Arkin A.P., Lim W.A. (2013). Repurposing CRISPR as an RNA-Guided Platform for Sequence-Specific Control of Gene Expression. Cell.

